# Microfibres and macroscopic films from the coordination-driven hierarchical self-assembly of cylindrical micelles

**DOI:** 10.1038/ncomms12371

**Published:** 2016-08-19

**Authors:** David J. Lunn, Oliver E. C. Gould, George R. Whittell, Daniel P. Armstrong, Kenneth P. Mineart, Mitchell A. Winnik, Richard J. Spontak, Paul G. Pringle, Ian Manners

**Affiliations:** 1 School of Chemistry, University of Bristol, Bristol BS8 1TS, UK; 2 Department of Chemical and Biomolecular Engineering, North Carolina State University, Raleigh, North Carolina 27695, USA; 3 Department of Chemistry, University of Toronto, Toronto, Ontario, Canada M5S 3H6; 4 Department of Materials Science and Engineering, North Carolina State University, Raleigh, North Carolina 27695, USA

## Abstract

Anisotropic nanoparticles prepared from block copolymers are of growing importance as building blocks for the creation of synthetic hierarchical materials. However, the assembly of these structural units is generally limited to the use of amphiphilic interactions. Here we report a simple, reversible coordination-driven hierarchical self-assembly strategy for the preparation of micron-scale fibres and macroscopic films based on monodisperse cylindrical block copolymer micelles. Coordination of Pd(0) metal centres to phosphine ligands immobilized within the soluble coronas of block copolymer micelles is found to induce intermicelle crosslinking, affording stable linear fibres comprised of micelle subunits in a staggered arrangement. The mean length of the fibres can be varied by altering the micelle concentration, reaction stoichiometry or aspect ratio of the micelle building blocks. Furthermore, the fibres aggregate on drying to form robust, self-supporting macroscopic micelle-based thin films with useful mechanical properties that are analogous to crosslinked polymer networks, but on a longer length scale.

## Abstract (web summary)

Anisotropic nanoparticles made from block copolymers are important building blocks for synthetic hierarchical materials. Here, the authors report a reversible coordination-driven self-assembly strategy for the preparation of micron-scale fibres and macroscopic films based on monodisperse cylindrical micelles.

## Graphical abstract

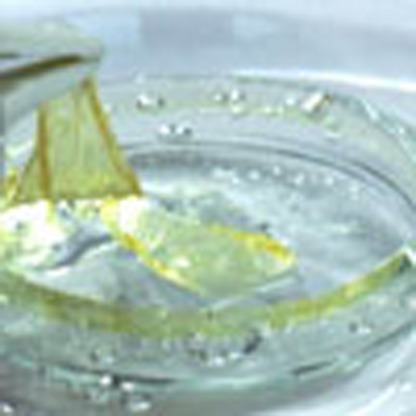

Nature is replete with examples of the bottom-up preparation of functional materials that display hierarchical order^1^,^2^,^3^,^4^. Metal coordination plays a vital role in many of these biological systems. For example, mammalian bone is reinforced by the reversible crosslinking of anisotropic collagen fibres with calcium ions^5^, and marine mussels derive their underwater adhesion and self-healing properties from the iron-mediated crosslinking of catechol groups^6^,^7^. Such metal–ligand interactions can be reversible and dynamic, with a wide range of bond strengths and labilities dependent on the choice of metal, ligand and coordination mode. In synthetic materials, metal coordination chemistry has been used for the preparation of a variety of supramolecular and covalent polymers^8^,^9^,^10^,^11^,^12^,^13^,^14^, dendrimers^15^,^16^, macrocycles^17^,^18^, gels^19^,^20^,^21^,^22^, metal-organic frameworks^23^,^24^ and protein assemblies^25^,^26^. However, the use of metal–ligand interactions is much less explored in the area of colloidal self-assembly. Metal coordination has been used to prepare hybrid magnetite/polystyrene nanoparticle chains through interparticle crosslinking after prior alignment of nanoparticles using an external magnetic field^27^. In addition, the coordination-driven self-assembly of colloidal particles has been described where immobilized palladium centres and pyridyl ligands were used to direct the interparticle linking of adjacent ‘patchy' polystyrene particles^28^.

The use of anisotropic building blocks is increasingly recognized as a key requirement for the realization of well-defined hierarchical structures^29^,^30^. Block copolymer (BCP) solution self-assembly is an elegant strategy by which such anisotropic subunits can be conveniently prepared in the form of colloidal, core-shell nanoparticles^31^,^32^,^33^,^34^,^35^. For example, BCP micelles bearing surface patches of segregated chemical functionality have been used as building blocks for precise hierarchical self-assembly under kinetic control to prepare complex mesostructures^36^,^37^. Furthermore, living crystallization-driven self-assembly (CDSA) of BCPs with a crystallizable core-forming block can be used to prepare monodisperse cylindrical micelles with controlled lengths and block co-micelles with compartmentalized chemical functionality^38^,^39^,^40^,^41^. On addition of a selective solvent, amphiphilic block co-micelle building blocks form ‘supermicelles', with structures ranging from micrometre-sized spherical or cylindrical assemblies to continuous one-dimensional or three-dimensional (3D) superlattices^42^,^43^,^44^. Cylindrical micelles have also been assembled end to end by the addition of further homopolymer with the same composition as the core-forming block. This additional homopolymer functions as a ‘glue' between micelle subunits, affording short, polydisperse, linear and branched chains of micelles^45^.

Despite these advances, methods for the self-assembly of anisotropic BCP nanoparticles are generally limited to the exploitation of building-block amphiphilicity^46^. Approaches that utilize alternative interactions are anticipated to diversify both the structures and properties of the resulting hierarchical materials, but very few examples have been reported thus far. Multiblock assemblies have been prepared by the covalent head-to-tail linking of BCP nanotubes, where coupling was achieved using carboxyl and amino groups that protruded from the termini of the precursors^47^. This approach has also been extended to prepare composite ‘nanoropes' by wrapping amino-containing nanocylinders around carboxyl-containing nanofibres, where the functionality was localized within the BCP micelle coronas^48^. Herein we demonstrate the reversible coordination-driven hierarchical self-assembly of monodisperse cylindrical BCP micelles, affording a series of complex superstructures and stable linear fibres that can be further processed into macroscopic micelle-based thin films.

## Results

### Preparation of monodisperse phosphine-containing BCP micelles

**Figure 1:**
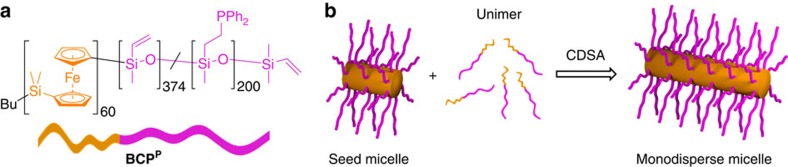
Preparation of monodisperse BCP^P^ micelles. Schematic depiction of (**a**) **BCP**^**P**^ and (**b**) the living CDSA of **BCP**^**P**^ in a selective solvent to afford monodisperse cylindrical micelles with a crystalline PFS core (orange) and a phosphine-functionalized polysiloxane corona (pink). The micelle illustrations in this figure are of idealized structures. The polysiloxane corona presumably overhangs at the core termini to provide some ‘dynamic' coverage of the crystal face.

The BCP poly(ferrocenyldimethylsilane)_60_-*b*-poly(methylvinylsiloxane)_574_ (PFS_60_-*b*-PMVS_574_), containing vinyl groups suitable for the incorporation of pendant functional groups, was prepared by sequential living anionic polymerization. Radical hydrophosphination under photolytic conditions was used to functionalize a percentage of the vinyl groups with diphenylphosphine, affording **BCP**^**P**^ (Fig. 1, Supplementary Fig. 1 and Supplementary Table 1) with 35% -PPh_2_ incorporation based on analysis by proton nuclear magnetic resonance (^1^H NMR) spectroscopy. Monodisperse cylindrical micelles were prepared from **BCP**^**P**^ by living CDSA utilizing the crystalline nature of the PFS core-forming block^38^,^39^,^40^,^41^. First, polydisperse cylindrical micelles were prepared by the homogeneous nucleation of **BCP**^**P**^ in EtOAc, a selective solvent for the polysiloxane block (Supplementary Fig. 2). The resulting micelles were then sonicated to yield short seed micelles (*L*_n_=37 nm, *L*_w_/*L*_n_=1.16, Supplementary Fig. 3) suitable for the initiation of living CDSA. Monodisperse **BCP**^**P**^ micelles (**M**_**545**_: *L*_n_=545 nm, *L*_w_/*L*_n_=1.05) were subsequently prepared by the living CDSA of a known amount of **BCP**^**P**^ unimer (molecularly dissolved BCP in a good solvent for both blocks) from the termini of the seed micelles. By changing the unimer-to-seed ratio, the lengths and therefore aspect ratios of the resulting micelles could be precisely controlled to prepare a variety of suitable micelle-building blocks (Supplementary Fig. 4 and Supplementary Table 2).

### Coordination-driven hierarchical self-assembly

Complexation of the phosphine ligands embedded in the micelle corona by Pd(II) centres was explored by the addition of varying amounts of Pd(OAc)_2_ to solutions of **M**_**545**_ in EtOAc. Transmission electron microscopy (TEM), energy dispersive X-ray spectroscopy (EDX) and ^31^P NMR spectroscopy confirmed the metal coordination and structural integrity of the cylindrical micelles (Supplementary Figs 5 and 6). After metal complexation, the resulting BCP cylinders could be dispersed in a good solvent for both blocks (for example, tetrahydrofuran (THF)) without dissolution, consistent with intramicellar coronal crosslinking (Supplementary Figs 7 and 8). Presumably, the immobilized monodentate phosphine ligands or vinyl groups from neighbouring polymer chains within the micelle coronas coordinate to the same Pd(II) metal centre.

**Figure 2:**
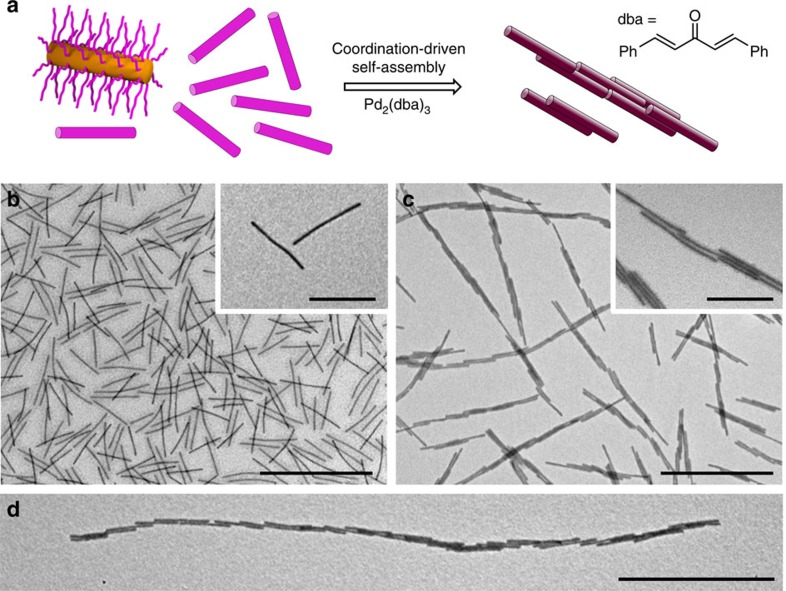
Coordination-driven hierarchical self-assembly of cylindrical BCP micelles. (**a**) Schematic depiction of the coordination-driven self-assembly of **BCP**^**P**^ micelles on addition of Pd_2_(dba)_3_. Pink and purple blocks correspond to **BCP**^**P**^ and Pd-coordinated **BCP**^**P**^, respectively. TEM images of **M**_**545**_ (0.5 mg ml^−1^) before Pd_2_(dba)_3_ addition (**b**), linear fibres of **M**_**545**_ (0.5 mg ml^−1^) after Pd_2_(dba)_3_ addition (**c**) and >10 μm linear fibre of **M**_**545**_ after Pd_2_(dba)_3_ addition and further dilution with EtOAc (**d**) (see Supplementary Fig. 11 for a lower-magnification image). Scale bars correspond to 2,000 nm in main images and 500 nm in insets.

We also investigated the complexation of Pd(0) centres by adding 0.5 equiv. Pd(0) as Pd_2_(dba)_3_ (dba, dibenzylideneacetone) to a solution of **M**_**545**_ in EtOAc. In contrast, aggregation of the cylindrical micelles in this case afforded long linear fibres of connected cylindrical micelles, as observed by TEM analysis after solvent evaporation (Fig. 2). This was consistent with considerable inter-, rather than solely intra-, micellar coordination. The resulting structures were analysed by EDX and ^31^P NMR spectroscopy (Supplementary Figs 5 and 9). To confirm that the linear fibres observed were not simply the result of a drying effect during TEM sample preparation, the solutions were likewise examined by dynamic light scattering (DLS) before solvent evaporation (Supplementary Fig. 10). This indicated the formation of much larger aggregates in solution in the case of the addition of Pd(0) centres, whereas no significant change in hydrodynamic size was detected when Pd(II) was added, consistent with intramicellar coordination in the latter case.

When the solution of the Pd(0) species Pd_2_(dba)_3_ was added to a solution of **BCP**^**P**^ micelles in EtOAc, the initial red colour faded over several minutes, presumably as the immobilized phosphines within the micelle coronas displaced the coordinated dba ligands. The resulting fibres formed by micelle aggregation remained colloidally stable and the solution could be diluted with negligible change in their length. However, the addition of a more strongly coordinating phosphine could be used to remove the intermicelle crosslinks and disassemble the linear fibres into their constituent micelle subunits. For example, when an excess of the chelating phosphine 1,2-bis(diphenylphosphino)ethane (dppe) was added to a solution of linear fibres at ambient temperature, the solution became yellow, consistent with the formation of Pd(dppe)_2_, as confirmed by ^31^P NMR spectroscopy. Subsequent TEM analysis in this case revealed the regeneration of discrete, non-aggregated cylindrical micelles, confirming the reversibility of the coordination-driven self-assembly (Supplementary Fig. 12).

The divergent behaviour of the **BCP**^**P**^ cylindrical micelles on addition of Pd(II) and Pd(0) centres presumably reflects the different coordination preferences for the metal centres in these two oxidation states. The coronas of **BCP**^**P**^ cylindrical micelles represent an unusual and complex coordination environment for both metal centres. Intramicelle crosslinking is anticipated to occur if a metal centre coordinated to a phosphine ligand on one polymer forms a bond with a phosphine located in close proximity on a neighbouring polymer chain (Supplementary Figs 7 and 13). In the corona of a micelle, the main coordination scenarios for Pd(OAc)_2_ would be expected to be Pd(L_1_)(-PPh_2_)(OAc)_2_ and Pd(-PPh_2_)_2_(OAc)_2_ (ref. 49), where L_1_ would most likely correspond to free vinyl groups along the polymer chain (Supplementary Figs 14 and 15) or bridging acetate ligands. In contrast, the complexes resulting from the addition of Pd_2_(dba)_3_ are expected to consist of Pd(dba)(-PPh_2_)_2_ in dynamic equilibrium with Pd(vinyl)(-PPh_2_)_2_ and Pd(-PPh_2_)_3_ environments, the latter involving three phosphine ligands^50^. As the micelle corona possesses a phosphine loading of only 35%, there may be, on average, only two readily available phosphines for the coordination sphere of each metal centre. In the case of the Pd(II) species Pd(OAc)_2_, this would result in intramicelle crosslinking. However, for the Pd(0) precursor Pd_2_(dba)_3_, metal centres at the periphery of micelle coronas would be available to take advantage of intermicelle collisions, thereby forming complexes with more than two phosphine ligands and causing intermicellar crosslinking.

### Fibre length variation using coordination-driven hierarchical self-assembly

**Figure 3:**
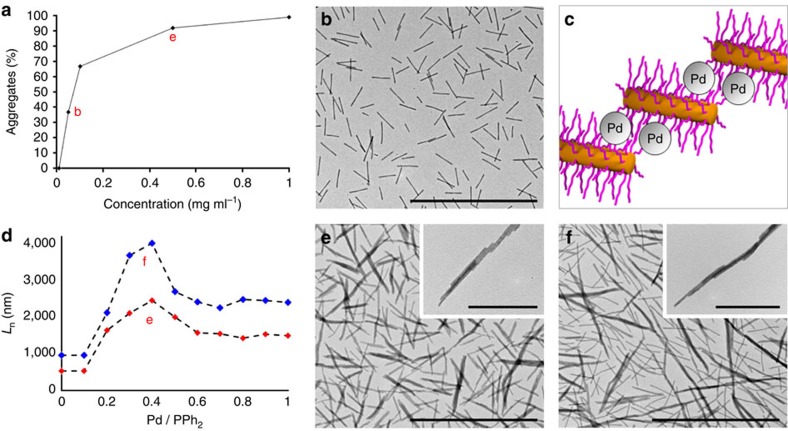
Controlled coordination-driven hierarchical self-assembly. (**a**) Dependence of the percentage of micelles that aggregate into linear fibres on initial micelle concentration, 24 h after addition of Pd_2_(dba)_3_ (0.4 equiv. Pd(0)). Labelled points (**b**,**e**) correspond to TEM images (**b**,**e**), respectively. (**b**) TEM image of **M**_**545**_ (0.05 mg ml^−1^) 24 h after addition of Pd_2_(dba)_3_. (**c**) Schematic depiction of the intermicelle crosslinking between the coronas of two adjacent but offset micelles. (**d**) Mean fibre length (*L*_n_) as a function of normalized Pd(0) concentration for the coordination-driven hierarchical self-assembly of cylindrical **BCP**^**P**^ micelle subunits **M**_**545**_ (*L*_n_=545 nm, *L*_w_/*L*_n_=1.05; red data points) and **M**_**970**_ (*L*_n_=970 nm, *L*_w_/*L*_n_=1.04; blue data points) at 0.5 mg ml^−1^ in EtOAc (see Supplementary Fig. 21 for an enlarged graph). Labelled points (**e**,**f**) correspond to TEM images (**e**,**f**), respectively. (**e**) TEM images of **M**_**545**_ (0.5 mg ml^−1^) 24 h after addition of Pd_2_(dba)_3_ (0.4 equiv. Pd(0)). (**f**) TEM images of **M**_**970**_ (0.5 mg ml^−1^) 24 h after addition of Pd_2_(dba)_3_ (0.4 equiv. Pd(0)). Scale bars correspond to 5,000 nm in main images and 1,000 nm in insets.

The concentration of **BCP**^**P**^ micelles was observed to have a significant influence on the degree of aggregation and length of linear fibres obtained by coordination-driven self-assembly (Fig. 3a). At a lower concentration of 1 mg ml^−1^, 24 h after Pd_2_(dba)_3_ addition, 99% of the micelles were involved in coordination-driven self-assembly. As the micelle concentration was decreased, the percentage of micelle subunits that coordinated to form linear fibres decreased until negligible self-assembly was observed below a concentration of 0.05 mg ml^−1^ (Fig. 3b). At these very low micelle concentrations, predominantly intra- rather than intermicelle crosslinking occurs, as evidenced by the stability of the BCP cylinders when dispersed in a good solvent for both blocks (Supplementary Fig. 13). These dilute micelle solutions remained stable indefinitely after the addition of Pd(0) with negligible change in the number micelles involved in intermicelle crosslinking. Notably, longer fibres were only obtained at higher concentrations when more of the micelles were involved in intermicelle crosslinking. At micelle concentrations >1 mg ml^−1^ some precipitation of the larger fibre-like micelle aggregates was observed. Moreover, when solutions of **BCP**^**P**^ micelles were stirred immediately after the addition of Pd(0) to increase the number of micelle collisions, almost complete aggregation and precipitation of the micelles occurred. In a control experiment, Pd_2_(dba)_3_ was added to solutions of non-phosphine-containing micelles (Supplementary Figs 16 and 17) and no coordination-driven self-assembly was observed over the time periods required for the controlled aggregation of **BCP**^**P**^-containing micelles.

To quantify the effect of stoichiometry on coordination-driven self-assembly, different amounts of a Pd_2_(dba)_3_ stock solution (0.1–1 equiv. Pd(0) with respect to immobilized phosphine) were added to solutions of **M**_**545**_ (0.5 mg ml^−l^) in EtOAc. On the basis of our initial experiments, a micelle concentration of 0.5 mg ml^−1^ was chosen at which ca. 92% of the micelles participate in coordination-driven self-assembly (Fig. 3a), resulting in fibres that are sufficiently long to be readily detected, but not so long as to make accurate analysis by TEM difficult (where fibres span several grid squares). Statistical analysis of TEM images obtained from samples drop-cast after 24 h (Supplementary Fig. 18 and Supplementary Table 3) revealed a trend between the mean length (*L*_n_) of the coordination structures and the Pd(0):phosphine ratio (Fig. 3d). The maximum mean length of the linear fibres was achieved on addition of 0.4 equiv. of Pd(0): *L*_n_=2,470 nm, *L*_w_/*L*_n_=1.18 (Fig. 3e).

To investigate the influence of micelle length on coordination-driven self-assembly, longer cylindrical **BCP**^**P**^ micelles (**M**_**970**_: *L*_n_=970 nm, *L*_w_/*L*_n_=1.04) were prepared. Different amounts of a Pd_2_(dba)_3_ stock solution (0.1–1 equiv. Pd(0) with respect to immobilized phosphine) were then added to solutions of **M**_**970**_ (0.5 mg ml^−1^) in EtOAc and the solutions similarly aged for 24 h. Statistical analysis of TEM images indicated an analogous trend to that observed with the shorter **M**_**545**_ micelles (Fig. 3d) although, as expected, larger mean lengths were observed due to the increased length of the micelle-building blocks (Supplementary Fig. 19 and Supplementary Table 3). Once again a maximum mean length was obtained when 0.4 equiv. of Pd(0) was added: *L*_n_=4,030 nm, *L*_w_/*L*_n_=1.24 (Fig. 3f). When more than 1 equiv. of Pd(0) was added to solutions of either **M**_**545**_ or **M**_**970**_, both side-to-side and end-to-end aggregation were detected, which led to the precipitation of a large number of the fibres (Supplementary Fig. 20).

The overall dimensions of the resulting fibres are presumably governed by a complex process that involves competing intra- and intermicelle crosslinking by Pd(0) coordination. Once several intermicelle crosslinks are established between adjacent micelles during the initial coordination phase of Pd(0) to **BCP**^**P**^ micelles, the resulting aggregate is likely to be kinetically trapped. As a consequence of this trapping effect together with the high aspect ratio of the cylindrical micelle building blocks, the majority of micelles would be expected to aggregate offset from one another (Fig. 3c). This explains the observation of a predominantly linear extension of the resulting fibres rather than lateral growth to yield two-dimensional structures. Higher micelle concentrations would be expected to favour larger degrees of aggregation and therefore the formation of longer micelles, as was detected (Fig. 3a). The Pd(0):phosphine ratio also has a substantial influence on the fibre length, but the relationship is more complex. On one hand, increased amounts of Pd(0) would be expected to favour intermicelle crosslinking and micelle aggregation, and should therefore increase fibre length. However, to form an intermicelle crosslink a Pd(0) centre must bind at least two phosphine groups from different micelles. At higher Pd(0):phosphine ratios the probability of two phosphines being available to coordinate to Pd(0) would decrease, thereby favouring a lower degree of aggregation. As a result of these two competing effects, a maximum in a plot of fibre length versus Pd(0):phosphine ratio is anticipated, and in fact, observed (Fig. 3d).

### Complex superstructures by hierarchical self-assembly

**Figure 4:**
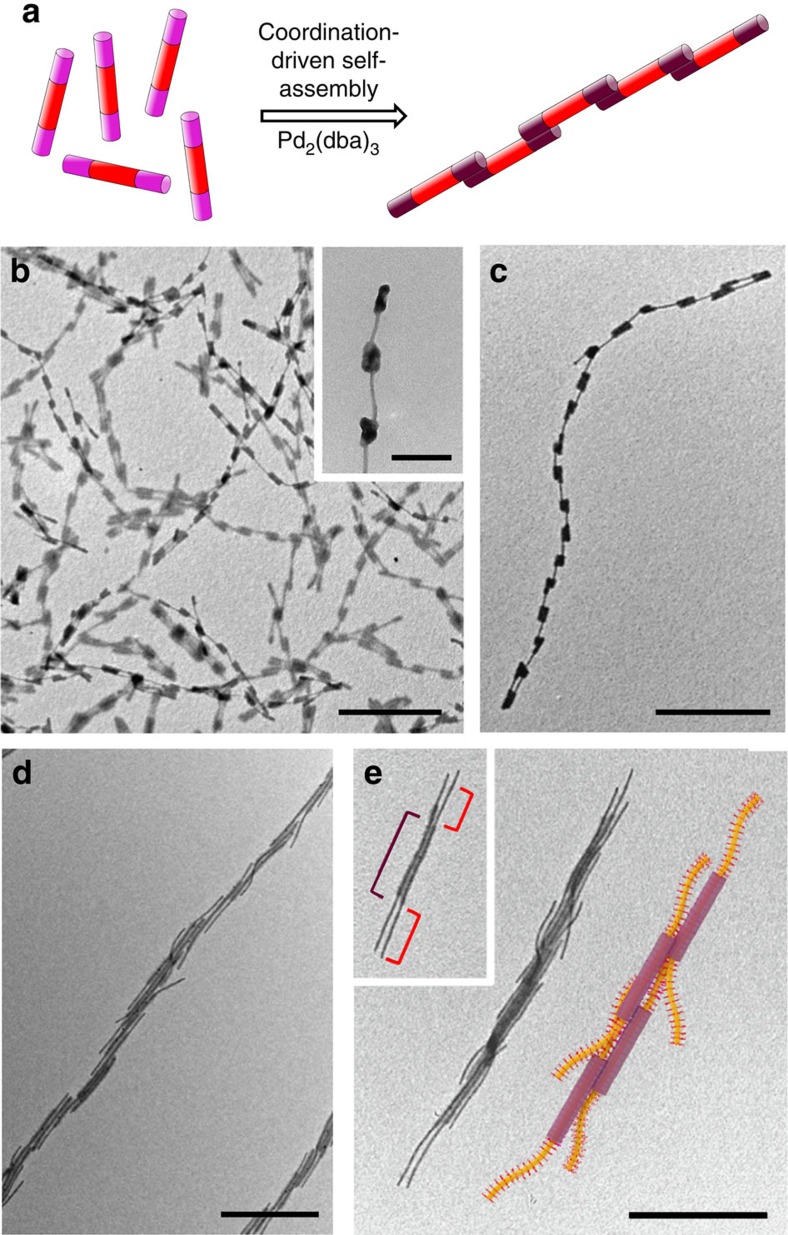
Complex superstructures by hierarchical self-assembly. (**a**) Schematic depiction of the coordination-driven self-assembly process. Red, pink and purple blocks in schematics correspond to PFS-*b*-PDMS, **BCP**^**P**^ and Pd-coordinated **BCP**^**P**^, respectively. TEM images of A–B–A triblock co-micelles (*L*_n_=215 nm, *L*_w_=220 nm, *L*_w_/*L*_n_=1.03) after coordination of Pd_2_(dba)_3_ (0.4 equiv. Pd(0)) at a micelle concentration of 1 mg ml^−1^ to favour longer multicompartment chains (**b**) and after dilution to 0.1 mg ml^−1^ (**c**). (**d**,**e**) TEM images of branched nanostructures 24 h after the addition of PFS_63_-*b*-PDMS_513_ (denoted by red bracket in inset) to a solution of linear coordinated fibres (denoted by purple bracket in inset). A schematic representation of the hairy rod-like superstructures is shown in **e**. Scale bars correspond to 1,000 nm in main images and 200 nm in inset.

Recent work has shown that amphiphilic block co-micelles can be used as building blocks for the assembly of a range of hierarchical superstructures^36^,^37^,^42^,^43^,^44^. The coordination-driven self-assembly strategy for cylindrical **BCP**^**P**^ micelles described here can be extended to the assembly of block co-micelles of low amphiphilicity containing spatially segregated regions of coronal chemistry. For example, A–B–A triblock co-micelles (*L*_n_=215 nm, *L*_w_/*L*_n_=1.03) with terminal A blocks of **BCP**^**P**^ and a central B block of PFS-*b*-PDMS (PDMS, polydimethylsiloxane), prepared by living CDSA via the addition of **BCP**^**P**^ to PFS-*b*-PDMS seeds (*L*_n_ ∼150 nm), also afford long linear fibres as observed by TEM after the addition of Pd_2_(dba)_3_. However, in these cases the degree of side-by-side aggregation appears markedly reduced so that essentially linear chains of micelles develop (Fig. 4a–c and Supplementary Fig. 22). When A–B–A triblock co-micelles (*L*_n_=465 nm, *L*_w_/*L*_n_=1.04) were prepared with a longer central PFS-*b*-PDMS segment (*L*_n_∼400 nm) and shorter **BCP**^**P**^ end blocks (*L*_n_∼30 nm), short linear and branched single chains of micelles, as well as cyclic aggregates, were observed (Supplementary Fig. 23). These results indicate that replacement of the phosphine-containing corona with regions of non-coordinating PDMS reduces the likelihood of side-by-side aggregation of the micelle-building blocks. Finally, B–A–B micelles were prepared (*L*_n_=360 nm, *L*_w_/*L*_n_=1.05), wherein the phosphine functionality was spatially isolated within the corona of the central A blocks (*L*_n_∼300 nm). In this case, as a result of the terminal PFS-*b*-PDMS B blocks lacking phosphine functionality, the formation of linear fibres became unfavourable and, on addition of Pd_2_(dba)_3_, coordination-driven assembly yielded stacked structures in which aggregation proceeded perpendicular to the central A block (Supplementary Fig. 24). Both the concentration of micelles and stoichiometry were observed to have an influence on the final dimensions of the resulting structures, albeit to a lesser extent than in the experiments using pure **BCP**^**P**^ micelles.

One of the principal advantages of this type of coordination-driven self-assembly is that hierarchical superstructures can be prepared by the use of orthogonal self-assembly techniques. For example, when further PFS-based BCP was added to a solution of linear fibres of **BCP**^**P**^ micelles crosslinked with Pd(0), additional growth occurred from the exposed micelle termini by living CDSA to generate hairy rod-like superstructures observable by TEM (Fig. 4d,e and Supplementary Fig. 25). Owing to the living nature of CDSA, the lengths of the supermicelle arms are dependent on the ratio of pre-existing Pd-coordinated micelles to added unimer. As a relatively low level of metal loading is required for coordination-driven hierarchical self-assembly, the degree of crosslinking and the length of the coronal segments appear to be insufficient to block the termini of the cylindrical micelles and prevent further growth, as has been previously observed^42^.

### Micelle-based thin films

The preparation of porous hybrid materials and nematic hydrogels^22^ using micelles has been reported^51^,^52^, although, to the best of our knowledge, no macroscopic materials have been prepared entirely from BCP micelles via hierarchical assembly. When coordination-driven self-assembly was attempted with a solution of **BCP**^**P**^ micelles in EtOAc with a concentration >1 mg ml^−1^, significant aggregation and precipitation of the micelles occurred (Supplementary Fig. 26). The suspensions were shaken to disperse the precipitated material, and drop-cast samples were observed by optical microscopy and TEM (Supplementary Fig. 27). Both techniques revealed that the aggregated material contained regions of interwoven fibres comprised of coordinated micelles.

**Figure 5:**
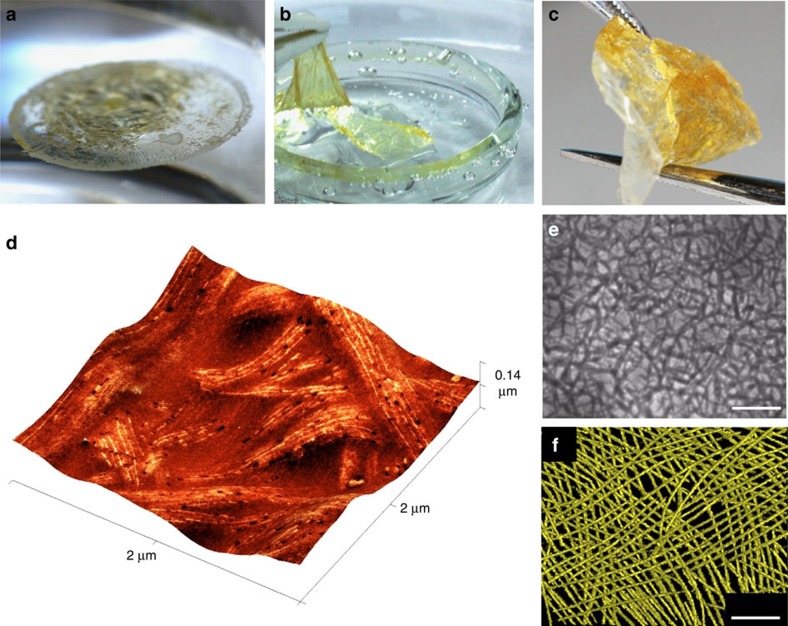
Micelle-based thin films. Photographic images of a micelle film prepared by drying a sample of linear fibres of coordinated **M**_**970**_; floating on water (**a**), in the process of being removed from the water surface (**b**) and being manipulated in air (**c**). AFM image displaying phase information superimposed on topographical height data (**d**), bright-field optical microscopy image (**e**) and rendered 3D image from TEMT (**f**) of a drop-cast sample of the solution of linear fibres of coordinated **M**_**970**_. Scale bars correspond to 10 and 0.2μm in **e** and **f**, respectively.

As the fibres described here are strengthened by intramicelle metal crosslinking, they represent promising stable building blocks for the bottom-up preparation of micelle-based materials. To prepare uniform bulk micelle films, an EtOAc solution of linear fibres of coordinated **BCP**^**P**^ micelles was layered on top of deionized water in a circular glass beaker. The organic solvent was allowed to evaporate, which led to the formation of a thin, self-supporting film at the water–air interface (Fig. 5a). The micelle film was subsequently removed and manipulated in air (Fig. 5b,c). The thickness of the film could be varied by changing the ratio of the amount of micelle material to the diameter of the container used, affording materials that varied from pale yellow for thin samples to orange for thicker examples (Supplementary Fig. 28). According to nanoindentation measurements (Supplementary Fig. 29), the hardness and Young's modulus of drop-cast films were determined to be 192.5±3.9 MPa and 1.2±0.4 GPa, respectively. For calibration, these values are in the approximate range expected for polymeric thermoplastic materials.

To prepare micelle-based films suitable for morphological and topological analyses, solutions of Pd-coordinated fibres of **M**_**970**_ were drop-cast and the resulting samples were subsequently investigated by atomic force microscopy (AFM), scanning electron microscopy (SEM), optical microscopy and transmission electron microtomography (TEMT)^53^,^54^ after solvent evaporation. Regions of interwoven coordinated fibres could be clearly observed by all four techniques (Fig. 5d–f and Supplementary Fig. 30). Significantly, three levels of hierarchy are evident by AFM (Fig. 5d and Supplementary Fig. 31); the bulk network material, the interwoven coordinated fibres and the individual **BCP**^**P**^ micelles. Moreover, TEMT analysis allowed for the rendering of a 3D image of the hierarchical material, wherein the aligned iron-rich micelle cores were clearly observed (Fig. 5f and Supplementary Fig. 32).

## Discussion

In summary, we describe a reversible, coordination-driven hierarchical self-assembly strategy for cylindrical micelles that allows access to micelle-based microfibres and thin films. Monodisperse cylindrical BCP micelle subunits, with phosphine ligands selectively located within their coronas, form micrometre-length fibres of staggered cylindrical BCP micelle-building blocks on treatment with a source of Pd(0). The final fibre lengths can be systematically varied by changing the reaction stoichiometry, overall micelle concentration or aspect ratio of micelle subunits. Moreover, as a significant step towards the preparation of functional macroscopic micelle-based materials, samples of the coordinated linear fibres can be dried to yield robust, self-supporting thin films. As control exists over the dimensions of the starting BCPs, the micelles and their segments, and the linear fibres, modifications at each of these levels of structural hierarchy would be expected to significantly influence the morphology and properties of the resulting macroscopic materials. This presents an opportunity to fabricate robust soft-matter-based constructs for applications as electroactive fibres and films, membranes for separation, nanocomposite materials and as catalyst supports.

## Methods

### Equipment and materials

The BCPs poly(ferrocenyldimethylsilane)_63_-*b*-polydimethylsiloxane_513_ (PFS_63_-*b*-PDMS_513_), PFS_68_-*b*-PMVS_670_ (PMVS, poly(methylvinylsiloxane)) and PFS_60_-*b*-PMVS_574_ were prepared by literature procedures (Supplementary Table 1)^55^. All air- or moisture-sensitive reactions were carried out in oven (200 °C)-dried glassware under a positive nitrogen pressure using standard Schlenk techniques or in an MBraun MB150B-G glove box under argon. THF for living anionic polymerizations and photochemical reactions was pre-dried with sodium and distilled off sodium/benzophenone before use. Anhydrous solvents were obtained using a modified Grubbs system of alumina columns manufactured by Anhydrous Engineering, and degassed by bubbling nitrogen before use. Tetramethylethylenediamine and 1,3,5-trimethyl-1,3,5-trivinylcyclotrisiloxane (V_3_) were pre-dried with calcium hydride and distilled before use. Chlorodimethylvinylsilane and chlorotrimethylsilane were distilled before use. Hexamethylcyclotrisiloxane (D_3_) was dissolved in pentane and pre-dried with calcium hydride before being dried *in vacuo* and sublimed onto a −5 °C cold finger. Diaceto palladium(II) and bis(dibenzylideneacetone) palladium(0) were purchased from Sigma-Aldrich and used without further purification. Tris(dibenzylideneacteone) dipalladium(0) was purchased from Sigma-Aldrich, and purified by a literature procedure^56^. All other commercially available compounds were used without further purification. Standard laboratory solvents were purchased from VWR or Fisher Scientific. Solvents for self-assembly were filtered (polytetrafluoroethylene membrane with 0.45 μm pore size) before use. Ethyl acetate for the self-assembly of phosphine-containing polymers was pre-dried with CaH_2_, distilled, filtered and stored under argon. Deionized water was used for the preparation of micelle-based films.

### Polymer characterization

The molecular weights of the BCPs were determined by combining the *M*_n_ of PFS homopolymers from matrix-assisted laser desorption/ionization time of flight (MALDI-TOF) mass spectrometry measurements, with the block ratio obtained by ^1^H NMR spectroscopy. MALDI-TOF mass spectrometry measurements of PFS homopolymers were performed using a Bruker Ultraflextreme running in linear mode. Samples were prepared using a *trans*-2-[3-(4-*tert*-butylphenyl)-2-methyl-2-propenylidene]malononitrile matrix (20 mg ml^−1^ in THF) and the polymer sample (2 mg ml^−1^ in THF), mixed in a 10:1 (v/v) ratio. Approximately 1 μl of the mixed solution was deposited onto a stainless-steel sample plate and allowed to dry in air. The *M*_n_ of the PFS homopolymers obtained by gel permeation chromatography (GPC) using laser light scattering to determine the absolute molecular weights corresponded well with those obtained by MALDI-TOF mass spectrometry. Theoretical molecular weights for the functionalized BCPs were used for yield calculations. Polydispersity indices (PDI=*M*_w_/*M*_n_) of all the polymers were obtained by GPC using a Viscotek VE 2001 Triple-detector gel permeation chromatograph equipped with automatic sampler, pump, injector, inline degasser, column oven (30 °C), styrene/divinylbenzene columns (pore sizes of between 500 and 100,000 Å), VE 3580 refractometer, four-capillary differential viscometer, VE 3210 UV/Vis detector (*λ*=450 nm) and VE 270 dual-angle laser light-scattering detector (7° and 90°). THF stabilized with 0.025% butylated hydroxytoluene (Fisher) was used as the chromatography eluent, at a flow rate of 1.0 ml min^−1^. Samples were dissolved in the eluent (2 mg ml^−1^) and filtered (polytetrafluoroethylene (PTFE) membrane with 0.45 μm pore size) before analysis. Calibration of the detectors was performed using polystyrene standards (Viscotek). The percentage hydrophosphination of **BCP**^**P**^ was calculated by both ^31^P NMR and ^1^H NMR spectroscopy, either by comparison to an internal standard, or by measuring the reduction of signals corresponding to vinyl group protons and the emergence of peaks corresponding to the hydrophosphinated product, respectively.

### NMR spectroscopy

^1^H, ^13^C, ^29^Si and ^31^P NMR spectra were recorded using either a JEOL Eclipse 300 MHz, JEOL Eclipse 400 MHz, Varian VNMR 400 MHz or Varian VNMR 500 MHz spectrometer. The chemical shifts (*δ*) are reported in parts per million (p.p.m.) and the coupling constants (*J*) in Hertz (Hz). Chemical shifts were referenced to residual dichloromethane at *δ* 5.32 p.p.m. (^1^H), and benzene at *δ* 7.14 p.p.m. (^1^H) and *δ* 128.06 p.p.m. (^13^C). ^1^H NMR integrations for the polymers were calculated after setting the methyl protons (6H) of PFS to 378H for PFS_63_-*b*-PDMS_513_, 408H for PFS_68_-*b*-PMVS_670_ and 360H for PFS_60_-*b*-PMVS_574_.

### Photoirradiation

Photoirradiation experiments were carried out with Pyrex glass-filtered emission from a water-cooled 125 W medium-pressure mercury lamp (Photochemical Reactors Ltd.). Pyrex glass filters out emission at wavelengths below 300 nm. The emission lines of the mercury lamp were 577–579, 546, 436, 408–405, 366–365, 334, 313, 302, 297, 289, 280, 270, 265 and 254 nm. An ethylene glycol/deionized water bath in conjunction with a thermostat was used to maintain constant temperatures during photoirradiation.

### Ultrasonication

A Hielsher UP100H 100 W ultrasonic processor equipped with a titanium sonotrode was used to prepare seed micelles. For oxygen-sensitive samples, sonication was conducted under a positive nitrogen atmosphere.

### Transmission electron microscopy

Samples for TEM were prepared by drop-casting 5 μl of the micelle solution onto a carbon-coated copper grid on a piece of filter paper, to remove excess solvent. Bright-field TEM micrographs were obtained on a JEOL1200EX II microscope operating at 120 kV and equipped with an SIS MegaViewIII digital camera. Stained samples were prepared as described above, aged for 24 h and then exposed to OsO_4_ vapour for 16 h in a sealed Petri dish. For the statistical length analysis, images were analysed using the ImageJ software package developed at the US National Institute of Health. A minimum of 200 individual cylinders were carefully traced by hand to determine the contour length. From these data *L*_n_ and *L*_w_ of each sample of cylindrical micelles were calculated using equations (1) and (2), respectively (where *L* is the length of an object and *N* is the number of objects).


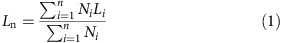



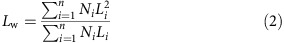


For the statistical length analysis of linear aggregates prepared by coordination-driven self-assembly, a minimum of 50 individual fibres were carefully traced by hand to determine the contour length. From these results *L*_n_ and *L*_w_ of each sample of linear aggregates were calculated using equations (1) and (2).

### EDX spectroscopic analysis

Complementary EDX measurements were conducted on a JEOL JEM 2010 microscope operated at 200 kV and equipped with a Kodak Electron Image Film SO-163 digital camera and an Oxford Instruments ISIS 310 system with silicon detector and ATW using a 35 nm spot size. All EDX experiments were conducted on the carbon-coated copper grids used for TEM.

### Bright-field optical microscopy

For the optical microscopy, sample solutions were drop-cast onto a glass slide. Imaging was carried out using a custom-built microscope with a 1.3 numerical aperture × 100 Plan-Neufluar, Zeiss objective lens. Movement of the field of view around the sample is achieved with a motorized x–y stage (MS2000, ASI) and a piezo-electric objective focussing system (Mipos 140 PL, Piezosystem Jena).

### Atomic force microscopy

Samples for AFM were prepared by spin-coating 10 μl of the micelle solution onto a freshly cleaved mica substrate or gold-coated mica. Tapping-mode images were obtained using a Multimode V atomic force microscope with a Nanoscope V controller. Nanosensors PPP NCHR10 cantilevers with a 10 nm rotated monolithic silicon tip were used. Imaging was conducted under ambient conditions. Images were analysed using Gwyddion, an open-source software programme.

### Scanning electron microscopy

Samples for SEM were prepared by drop-casting the sample solutions onto gold-coated mica substrates. Imaging was performed using a JEOL 5600LV SEM microscope.

### Dynamic light scattering

DLS measurements (173°) were performed using a Malvern Zetasizer Nano Series spectrometer equipped with a 633 nm red laser. Solutions of between 0.5 and 1 mg ml^−1^ were analysed in glass cuvettes at 20 °C. Dry and degassed ethyl acetate was used as the continuous phase for all experiments. In the case of the reversibility experiments, reproducible DLS data could not be obtained due to the strong colour of the molecular Pd(0) species formed during process.

### Nanoindentation

Hardness and modulus tests of thick cylindrical micelle films drop-cast onto silicon wafer were performed at ambient temperature on a Hysitron UBI-Nanoindenter equipped with a 90° conical diamond tip possessing a nominal 1 μm curvature radius (specifically designed for use with polymers). AFM was conducted in tapping mode to (i) map the surface features over a 10 × 10 μm^2^ specimen region and (ii) select areas with minimal surface roughness for indentation tests within the region of interest. Indentations were load-controlled at forces of 35, 50, 75, 100 and 150 μN, leading to penetration depths ranging from ca. 80 to 300 nm. Corresponding loading rates were applied at 16, 23, 35, 47 and 70 μN s^−1^, respectively, whereas unloading rates were maintained at the maximum load per second. Measurements from loading–unloading cycles such as the one provided in Supplementary Fig. 29 were replicated 11 times from different specimen areas. The hardness (*H*) of the drop-cast cylindrical micelle film from nanoindentation measurements is calculated from equation (3).


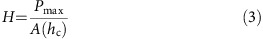


*P*_max_ denotes the pressure measured at the maximum penetration depth, *A* is the area of contact between the tip and the sample (calculated using an empirical tip area function based on the specific tip geometry), and *h*_c_ represents the contact depth. According to equation (3), *H* of the cylindrical micelle film is determined to be 192.5±3.9 MPa. Similarly, a reduced Young's modulus (*E*_r_) can likewise be determined from equation (4).


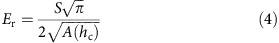


*S* is the stiffness discerned from the instantaneous change in load pressure at the point of unloading (that is, d*P*/d*h* evaluated at *h*_max_ identified in Supplementary Fig. 29). The Young's modulus of the micelle film (*E*) can then be computed from the reduced Young's modulus by knowing the modulus of the indentation tip (*E*_i_) and the corresponding Poisson's ratios (*υ* and *υ*_i_, respectively; equation (5)).


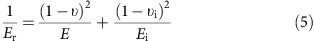


The values of *E*_i_ and *υ*_i_ used here for the diamond tip are 1,141 GPa and 0.07, respectively. Since Mott and Roland^57^ point out that many thermoplastics possess a Poisson's ratio between 0.3 and 0.5, we presume here that *υ*≈0.4, in which case the Young's modulus of the micelle film is 1.2 GPa (with a variation of ±0.4 GPa as *υ* changes by ±0.1). Because each copolymer molecule contains a substantial polysiloxane block, a Poisson's ratio approaching 0.5 is reasonable. The crystallizable block that regulates copolymer self-assembly and contributes to micelle stiffness is, however, likely to reduce the overall Poisson's ratio of the micelle film, as well as cause the plastic deformation evident in Supplementary Fig. 29.

### Low-voltage SEM

Backscattered-electron images of uncoated cylindrical micelle films drop-cast onto carbon-coated hexagonal grids were acquired by field-emission SEM performed on an ultrahigh-resolution FEI Verios 460l Schottkey emitter microscope operated at 1 kV.

### Transmission electron microtomography

3D TEMT images of cylindrical micelle films drop-cast onto carbon-coated hexagonal grids were generated from a series of TEM tilt images collected from −65° to +65° at an angular increment of 1.5° on a Gatan K2 Summit direct detection camera mounted on an automated FEI Tecnai F20 field-emission microscope operated at 200 kV. Image alignment was performed without the use of fiducial markers by modelling the high-precision goniometer^58^. The resulting aligned and mass-normalized image sets were subjected to reconstruction using the filtered (*r*-weighted) back-projection algorithm^59^, and 3D volume elements were produced by qualitative pixel thresholding.

### Preparation of **BCP** ^**P**^

Diphenylphosphine (2.00 ml, 2.14 g, 11.5 mmol) was added to a solution of PFDMS_61_-*b*-PMVS_574_ (0.46 g, 4.1 mmol vinyl groups) in dry THF (10 ml) under a nitrogen atmosphere. The reaction mixture was irradiated 3 cm from a mercury lamp at room temperature for 24 h. The solution was concentrated *in vacuo*, twice precipitated into degassed MeOH and dried *in vacuo* to afford the pure BCP, **BCP**^**P**^ (0.54 g, 74%, 35% hydrophosphination by ^1^H NMR) as an orange solid (Supplementary Fig. 1 and Supplementary Table 1). ^31^P NMR (202 MHz; C_6_D_6_): *δ* −9.4 (br s, -PPh_2_); ^31^P NMR (202 MHz; CDCl_3_): *δ* −9.1 (br s, -PPh_2_); ^31^P NMR (202 MHz; EtOAc): *δ* −8.7 (br s, -PPh_2_); ^1^H NMR (500 MHz; C_6_D_6_): *δ* 0.21–0.38 (1728H, br m, siloxane SiC*H*_*3*_), 0.56 (360H, s, Si(C*H*_*3*_)_2_), 0.89 (400H, br m, SiC*H*_*2*_), 2.29 (400H, br m, C*H*_*2*_P), 4.12 (240H, s, Cp*H*), 4.29 (240H, s, Cp*H*), 5.90–6.00 (750H, br m, CHC*H*_*2*_), 6.09–6.26 (375H, br m, C*H*CH_2_), 7.07–7.17 (1200H, br m, Ar*H*) and 7.54–7.57 (800H, br m, Ar*H*); ^13^C NMR (100 MHz, C_6_D_6_): *δ* −0.5 (Si(*C*H_3_)_2_), −0.1 (Si*C*H_3_), 13.6 (d, *J*=8.2 Hz, Si*C*H_2_), 21.4 (d, *J*=15.2 Hz, *C*H_2_P), 71.8 (2Cp*C*), 71.9 (Cp*C*Si), 73.7 (2Cp*C*), 128.7 (br s, Ar*C*), 133.3 (d, *J*=18.2 Hz, Ar*C*), 133.7 (br s, *C*HCH_2_), 137.1 (br s, CH*C*H_2_) and 139.8 (d, *J*=14.3 Hz, P*C*_ipso_). The polymer **BCP**^**P**^ was treated with sulphur (1 equiv.) in dry and degassed THF to oxidize the phosphine moieties before GPC analysis. ^31^P NMR (202 MHz; THF): *δ* 38.4 (br s, -P(S)Ph_2_).

### Preparation of cylindrical micelles

*General procedure*. In a sealed vial under an argon atmosphere, a solution of BCP in dry and degassed EtOAc (1 mg ml^−1^) was heated to 75 °C with stirring for 1 h. The pale yellow solution was allowed to cool and aged for 1 week. Polydisperse cylindrical micelles were observed by TEM analysis of the drop-cast solution (Supplementary Fig. 2).

### Preparation of **BCP** ^**P**^ seed micelles

A solution of polydisperse cylindrical micelles of BCP **BCP**^**P**^ in dry and degassed EtOAc (1 mg ml^−1^) under a nitrogen atmosphere was cooled to 0 °C and sonicated with a sonotrode at 50% power for 1 h to afford monodisperse seed micelles (*L*_n_=37 nm, *L*_w_=43 nm, *L*_w_/*L*_n_=1.16; Supplementary Fig. 3) as observed by TEM analysis. The same procedure was used for the preparation of monodisperse PFS_63_-*b*-PDMS_513_ seed micelles (*L*_n_=41 nm, *L*_w_=49 nm, *L*_w_/*L*_n_=1.19).

### Preparation of monodisperse **BCP** ^**P**^ micelles

*General procedure*. In a glove box under an argon atmosphere, 5, 10, 20 and 40 equiv. of **BCP**^**P**^ unimer (10 mg ml^−1^ in THF) were added to stirred stock solutions of monodisperse seed micelles diluted in dry and degassed EtOAc (to achieve a final **BCP**^**P**^ concentration of 1 mg ml^−1^). After addition the stirring was stopped and the mixtures aged for 1 week. Statistical length analysis of TEM images of the resulting nanostructures showed a linear growth dependence on the unimer-to-seed ratio (Supplementary Fig. 4 and Supplementary Table 2). In this study, negligible differences were observed between the CDSA behaviour of the non-phosphine-containing and phosphine-containing BCPs.

### Preparation of monodisperse A–B–A micelles

A is **BCP**^**P**^ and B is PFS-*b*-PDMS. In a glove box under an argon atmosphere, 200 μl PFS_63_-*b*-PDMS_513_ seeds (1 mg ml^−1^ in EtOAc) were diluted in 900 μl dry and degassed EtOAc. The seed solution was stirred and 20 μl of a PFS_63_-*b*-PDMS_513_ unimer solution (10 mg ml^−1^ in THF) was added dropwise. After addition the stirring was stopped and the mixture aged overnight. The micelle solution was stirred and 80 μl of a **BCP**^**P**^ unimer solution (10 mg ml^−1^ in THF) was added dropwise. After addition the stirring was stopped and the mixture aged for 1 week. Monodisperse cylindrical micelles were observed by TEM analysis of a drop-cast of the solution (*L*_n_=215 nm, *L*_w_=222 nm, *L*_w_/*L*_n_=1.03).

In a glove box under an argon atmosphere, 200 μl PFS_63_-*b*-PDMS_513_ seeds (1 mg ml^−1^ in EtOAc) were diluted in 900 μl dry and degassed EtOAc. The seed solution was stirred and 60 μl of a PFS_63_-*b*-PDMS_513_ unimer solution (10 mg ml^−1^ in THF) was added dropwise. After addition the stirring was stopped and the mixture aged overnight. The micelle solution was stirred and 40 μl of a **BCP**^**P**^ unimer solution (10 mg ml^−1^ in THF) was added dropwise. After addition the stirring was stopped and the mixture aged for 1 week. Monodisperse cylindrical micelles were observed by TEM analysis of a drop-cast of the solution (*L*_n_=465 nm, *L*_w_=484 nm, *L*_w_/*L*_n_=1.04).

### Preparation of monodisperse B–A–B micelles

A is **BCP**^**P**^ and B is PFS-*b*-PDMS. In a glove box under an argon atmosphere, 200 μl **BCP**^**P**^ seeds (1 mg ml^−1^ in EtOAc) were diluted in 2 ml dry and degassed EtOAc. The seed solution was stirred and 200 μl of a **BCP**^**P**^ unimer solution (10 mg ml^−1^ in THF) was added dropwise. After addition the stirring was stopped and the mixture aged for 1 week. The micelle solution was stirred and 20 μl of a PFS_63_-*b*-PDMS_513_ unimer solution (10 mg ml^−1^ in THF) was added dropwise. After addition the stirring was stopped and the mixture aged for 1 day. Monodisperse cylindrical micelles were observed by TEM analysis of a drop-cast of the solution (*L*_n_=360 nm, *L*_w_=378 nm, *L*_w_/*L*_n_=1.05).

### Coordination-driven self-assembly of cylindrical micelles

*General procedure using palladium(II) diacetate, Pd(OAc)_2_*. In a glove box under an argon atmosphere, a 0.002 M solution of Pd(OAc)_2_ in dry and degassed EtOAc (25 μl, 5 × 10^−5^ mmol, 0.5 equiv.) was added to a 0.5 mg ml^−1^ solution of **BCP**^**P**^ micelles in dry and degassed EtOAc (100 μl, 2 × 10^−4^ mmol·ml^−1^ -PPh_2_). The red solution was briefly shaken to mix and left to age for 24 h before analysis by ^31^P NMR spectroscopy (Supplementary Fig. 5), TEM and EDX (Supplementary Fig. 6). ^31^P NMR (202 MHz; EtOAc): *δ* 22.6 (br s, -PPh_2_). The micelles could be diluted with THF, a common solvent for both blocks, without disassembling (Supplementary Fig. 7). Samples of **BCP**^**P**^ micelles crosslinked with Pd(OAc)_2_ were not stable indefinitely and aggregation was observed when samples were analysed by TEM after 1 week (Supplementary Fig. 8).

*General procedure using tris(dibenzylideneacetone)dipalladium(0) chloroform adduct, Pd_2_(dba)_3_·CHCl_3_*. In a glove box under an argon atmosphere, a 0.002 M solution of Pd_2_(dba)_3_·CHCl_3_ in dry and degassed EtOAc (25 μl, 5 × 10^−5^ mmol, 0.5 equiv.) was added to a 0.5 mg ml^−1^ solution of **BCP**^**P**^ micelles in dry and degassed EtOAc (100 μl, 2 × 10^−4^ mmol·ml^−1^ -PPh_2_). The red solution was briefly shaken to mix and left to age for 24 h analysis by ^31^P NMR spectroscopy (Supplementary Fig. 5), TEM and EDX (Supplementary Fig. 9). ^31^P NMR (202 MHz; EtOAc): *δ* 22.9 (br s, -PPh_2_). See Supplementary note 1 for additional comments regarding the coordination-driven self-assembly.

### Reversibility of inter- and intramicelle crosslinking

*General procedure*. In a glove box under an argon atmosphere, a 0.01 M solution of 1,2-bis(diphenylphosphino)ethane (dppe) (100 μl, 1 × 10^−3^ mmol, 2 equiv. with respect to Pd) was added to a solution of **BCP**^**P**^ micelles (1 ml, 0.5 mg ml^−1^, 1 × 10^−3^ mmol -PPh_2_) crosslinked with Pd_2_(dba)_3_·CHCl_3_ (25 μl, 5 × 10^−5^ mmol) in dry and degassed EtOAc. The solution turned yellow on addition of dppe, characteristic of the formation of Pd(dppe)_2_, was shaken to mix and analysed by TEM and ^31^P NMR (Supplementary Fig. 12). ^31^P NMR (202 MHz; CD_2_Cl_2_): *δ* 31.1 (Pd(dppe)_2_). Notably, although dppe was strongly coordinating enough to remove intermicelle crosslinks, in the case of both Pd(II) and Pd(0) broad ^31^P NMR signals corresponding to Pd coordinated **BCP**^**P**^ at 30–32 p.p.m. were still observed (Supplementary Fig. 12).

### Preparation of hairy rod-like superstructures

*General procedure*. In a glove box under an argon atmosphere, different equivalents of PFS_63_-*b*-PDMS_513_ unimer (10 mg ml^−1^ in THF) were added to solutions of coordinated linear fibres of **BCP**^**P**^ micelles, diluted in dry and degassed EtOAc to maintain the THF percentage at ∼10% (Fig. 4d,e and Supplementary Fig. 25).

### Preparation of micelle-based materials

*General procedure*. In a glove box under an argon atmosphere, a 0.002 M solution of Pd_2_(dba)_3_·CHCl_3_ in dry and degassed EtOAc (25 μl, 5 × 10^−5^ mmol, 0.5 equiv.) was added to a 1 mg ml^−1^ solution of **BCP**^**P**^ micelles in dry and degassed EtOAc (100 μl, 2 × 10^−4^ mmol ml^−1^ -PPh_2_). The red solution was briefly shaken to mix and left to age for 24 h, over which time the colour faded to afford a cloudy pale yellow solution. The solution was transferred in air on to a layer of deionized water within a small glass Petri dish in the centre of a larger glass Petri dish. The EtOAc was evaporated with blowing nitrogen to afford a micelle film on the water surface. Deionized water was added slowly to float the film out of the smaller Petri dish where it could be removed from the surface and manipulated in air. The resulting material was analysed by optical microscopy, AFM, SEM and TEM. A sample of the film was dispersed by stirring in C_6_D_6_ and analysed by NMR. ^31^P NMR (202 MHz; C_6_D_6_): *δ* 29.9 (br s, -PPh_2_); ^1^H NMR (500 MHz; C_6_D_6_): *δ* 0.14–0.41 (1728H, br m, siloxane SiC*H*_*3*_), 0.56 (135H, s, Si(C*H*_*3*_)_2_), 2.50 (137H, br m, C*H*_*2*_P), 4.12 (100H, s, Cp*H*), 4.29 (95H, s, Cp*H*), 5.87–6.01 (319H, br m, CHC*H*_*2*_) and 6.16 (156H, br m, C*H*CH_2_). Signals corresponding to SiC*H*_*2*_ and the phosphine aryl protons could not be assigned due to peak broadening and overlap with adjacent signals. We hypothesize that the reduced integrals are due to the presence of remaining crosslinks between phosphine moieties that prevent complete dissolution of the material. Theoretical ^1^H NMR integrals are stated below. DLS confirmed the presence of aggregates in solution (Supplementary Fig. 26). Theoretical ^1^H NMR (500 MHz; C_6_D_6_): *δ* 0.21–0.38 (1728H, br m, siloxane SiC*H*_*3*_), 0.56 (360H, s, Si(C*H*_*3*_)_2_), 0.89 (400H, br m, SiC*H*_*2*_), 2.29 (400H, br m, C*H*_*2*_P), 4.12 (240H, s, Cp*H*), 4.29 (240H, s, Cp*H*), 5.90–6.00 (750H, br m, CHC*H*_*2*_), 6.09–6.26 (375H, br m, C*H*CH_2_), 7.07–7.17 (1200H, br m, Ar*H*) and 7.54–7.57 (800H, br m, Ar*H*).

### Data availability

The data that support the findings of this study are available from the corresponding authors on request.

## Additional information

**How to cite this article:** Lunn, D. J. *et al.* Microfibres and macroscopic films from the coordination-driven hierarchical self-assembly of cylindrical micelles. *Nat. Commun.* 7:12371 doi: 10.1038/ncomms12371 (2016).

## Supplementary Material

Supplementary Information Supplementary Figures 1-32, Supplementary Tables 1-3 and Supplementary Note 1
