# Supplementary material for: Microfibres and macroscopic films from the coordination-driven hierarchical self-assembly of cylindrical micelles
Source: Nat Commun. 2016 Aug 19;7:12371. doi: 10.1038/ncomms12371 (PMC4992161; doi:10.1038/ncomms12371)
Supplement: Supplementary Information — Supplementary Figures 1-32, Supplementary Tables 1-3 and Supplementary Note 1 [file ncomms12371-s1.pdf]

Supplementary figures

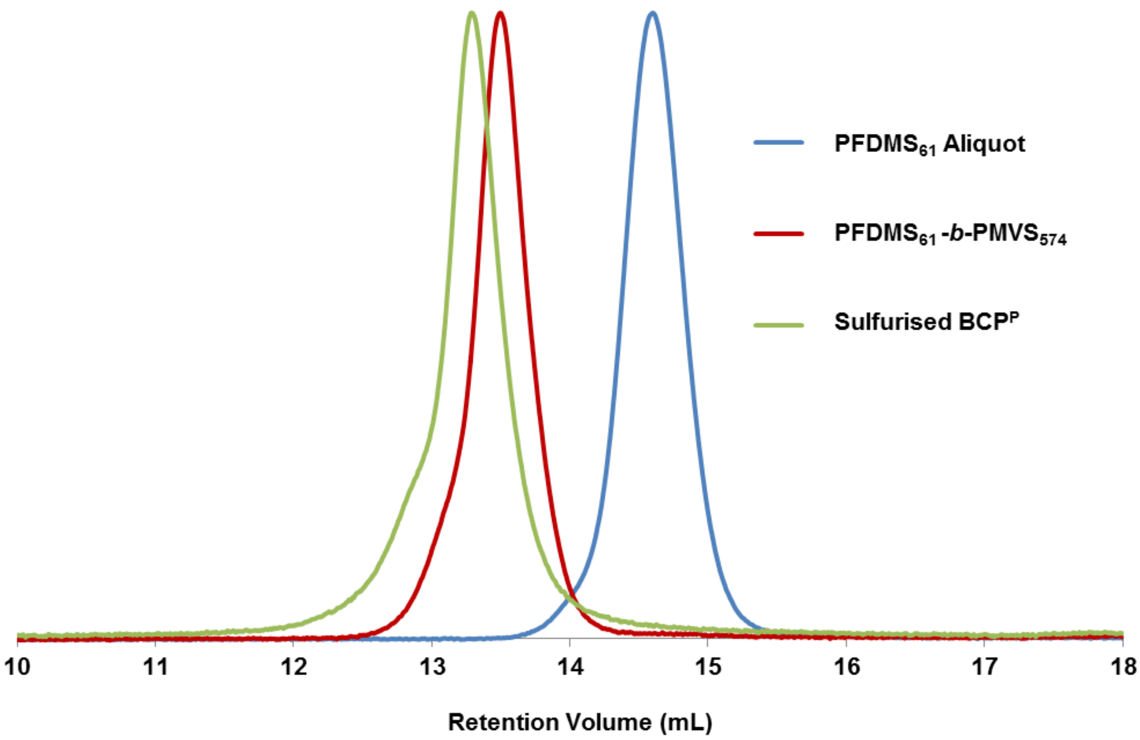

Supplementary Figure 1. Overlaid GPC traces (RI) of the $PFDMS_{61}$ aliquot, pure $PFDMS_{61}$-*b*-$PMVS_{574}$ copolymer and $\mathbf{BCP^P}$ after sulfurisation of pendant phosphines.

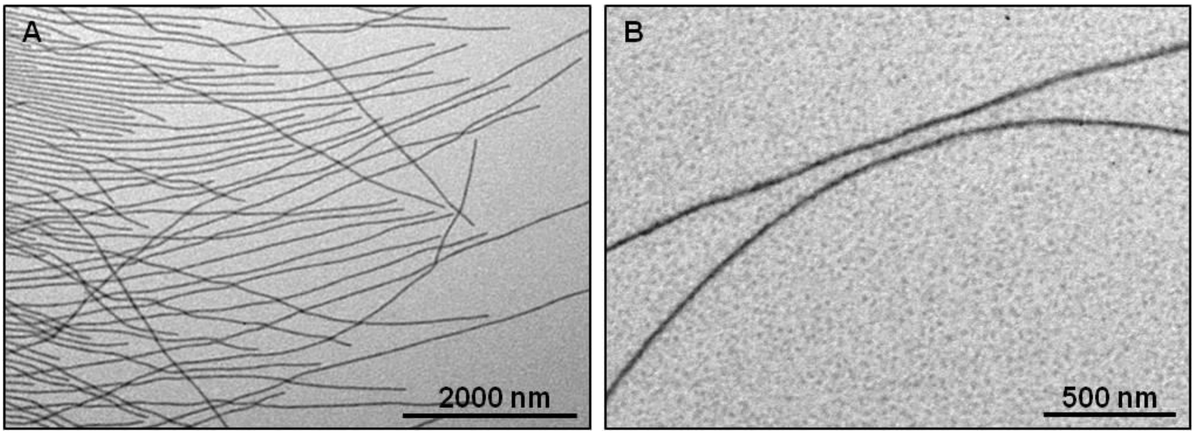

Supplementary Figure 2. TEM images of long (>10 µm) polydisperse **BCP**$^{\mathbf{P}}$ micelles (1 mg mL^{-1}) prepared by homogeneous nucleation in EtOAc after 1 week.

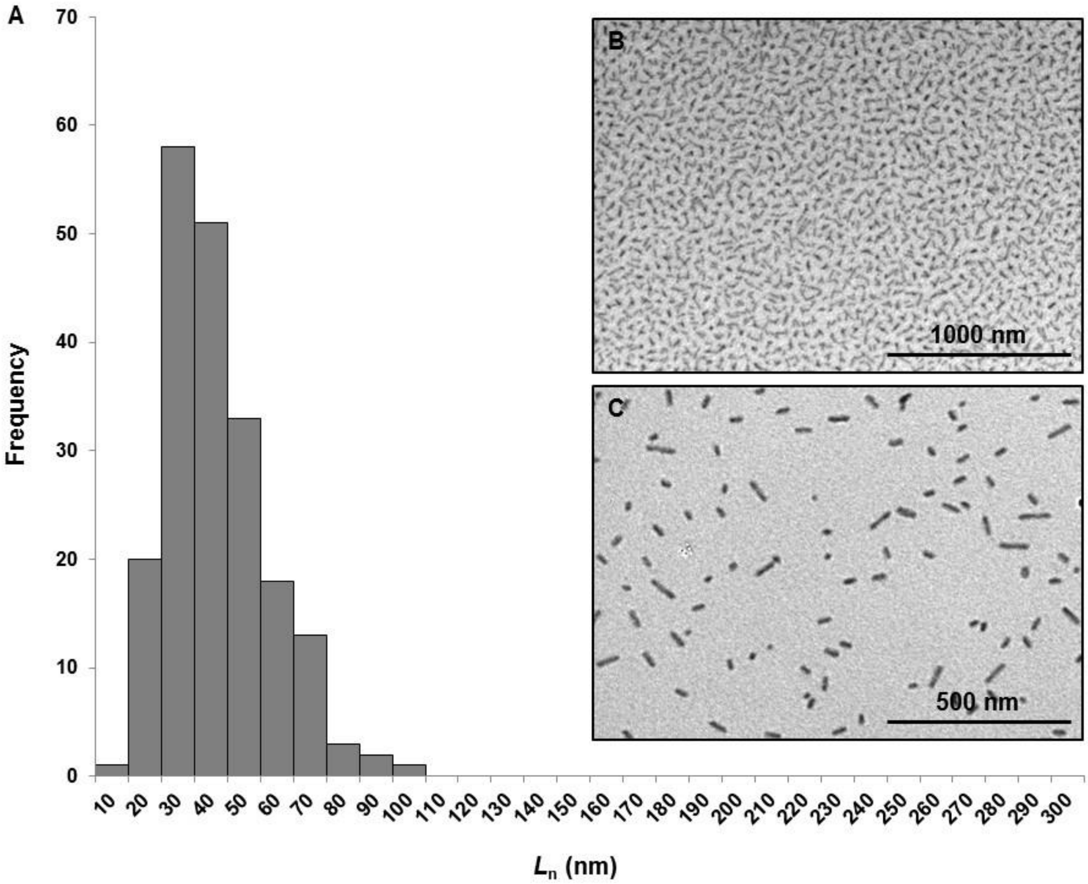

Supplementary Figure 3. Histogram of the contour length (L_n) distribution of **BCP^P** seed micelles (L_n = 37 nm, L_w = 43 nm, L_n/L_w = 1.16). **A and B)** TEM images of **BCP^P** seed micelles in EtOAc prepared by the sonication of long (>10 µm) polydisperse **BCP^P** micelles.

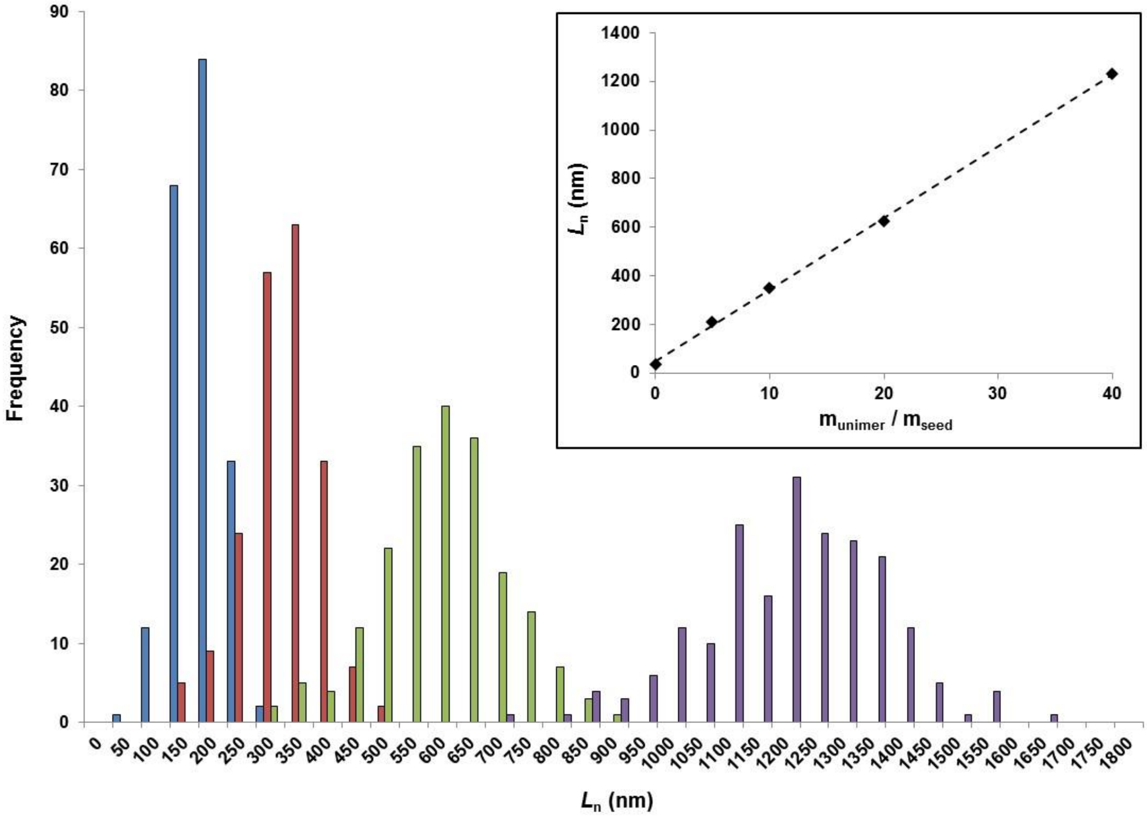

Supplementary Figure 4. Length distribution histograms for **BCP**$^{\mathbf{P}}$ micelles prepared by seeded growth in EtOAc. Inset: graph showing the linear dependence of the contour length (L_n) of **BCP**$^{\mathbf{P}}$ micelles on the unimer-to-seed ratio.

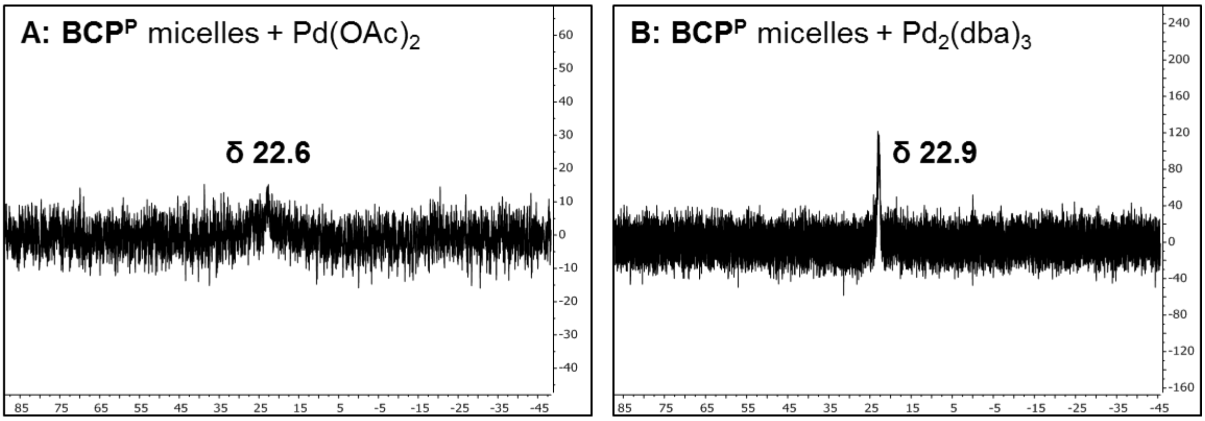

Supplementary Figure 5. ^{31}P NMR (202 MHz; EtOAc) spectra of **BCP^{P}** micelles after the addition of 0.5 equiv. of Pd as $Pd(OAc)_2$ (**A**) or $Pd_2(dba)_3$ (**B**). The broad peaks in both (**A**) and (**B**) are attributed to lability of the Pd-P linkages and the variety of potential coordination modes available for Pd(II) and Pd(0) metal centres within the micelle coronas. Additionally, the solubility of the micelle coronas, and thus corresponding NMR signals, are reduced with micelle crosslinking. The observed NMR spectra were obtained after several thousand scans (Varian VNMR 500 MHz spectrometer). For comparison, the precursor micelles possess a ^{31}P NMR peak at – 8.7 ppm in EtOAc corresponding to the uncoordinated phosphines.

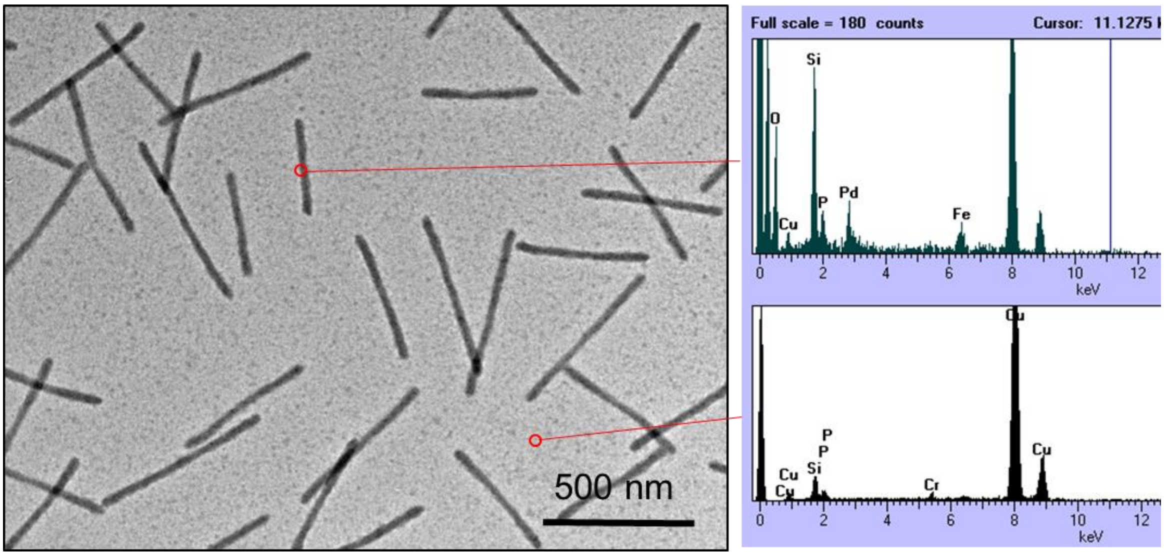

Supplementary Figure 6. TEM image and EDX analysis of **BCP^{P}** micelles in EtOAc after addition of 0.5 equiv. $Pd(OAc)_2$. Red spot denotes target area of EDX analysis (~35 nm diameter).

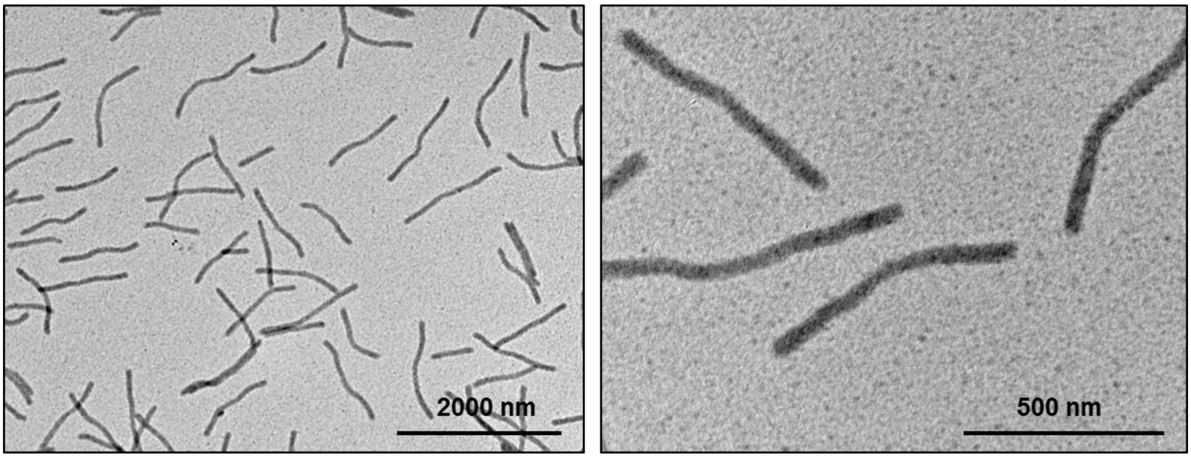

Supplementary Figure 7. TEM images of monodisperse $\mathbf{BCP^{P}}$ micelles dispersed in THF, a good solvent for both blocks, after intramicelle crosslinking with 0.5 equiv. $Pd(OAc)_2$.

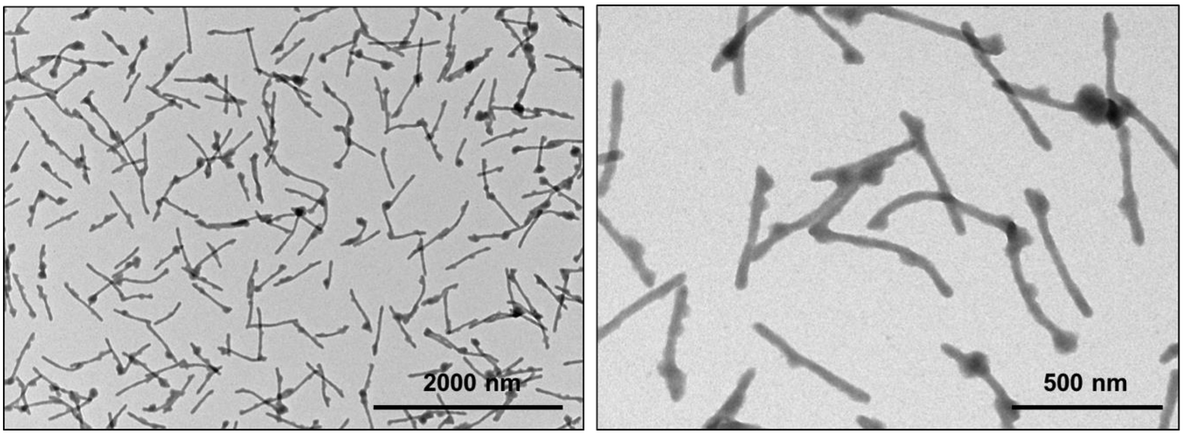

Supplementary Figure 8. TEM images of monodisperse **BCP^P** micelles in EtOAc one week after the addition of 0.5 equiv. $Pd(OAc)_2$. Micelle solutions were stored in a glove box under inert atmosphere.

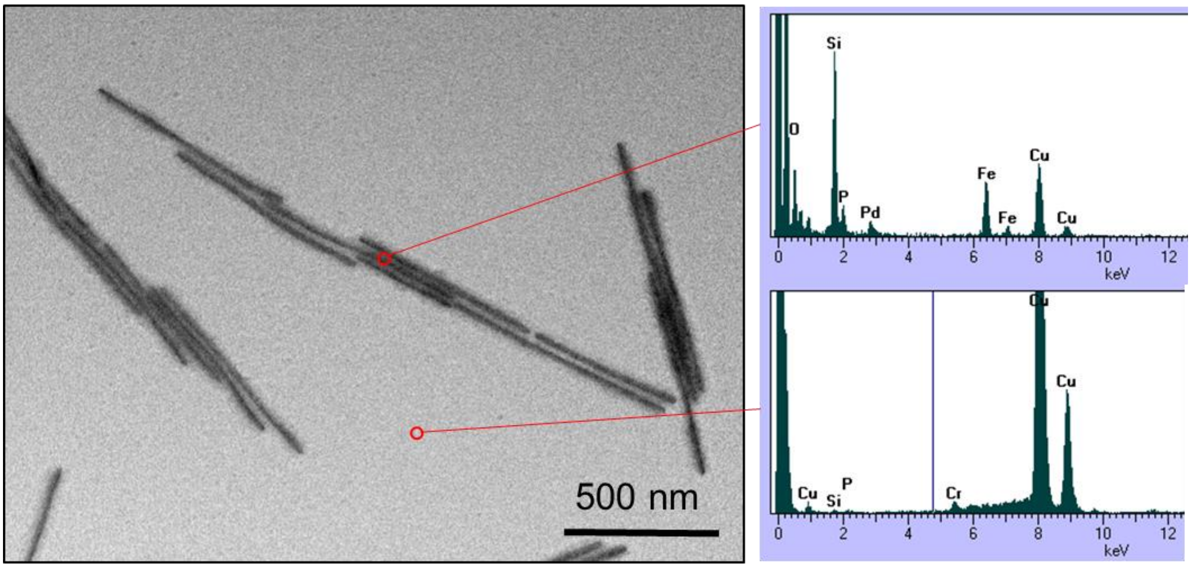

Supplementary Figure 9. TEM image and EDX analysis of **BCP**$^{\mathbf{P}}$ micelles in EtOAc after addition of $Pd_2(dba)_3$ (0.25 equiv. Pd(0)). The red circle denotes the target area for EDX analysis (~35 nm diameter).

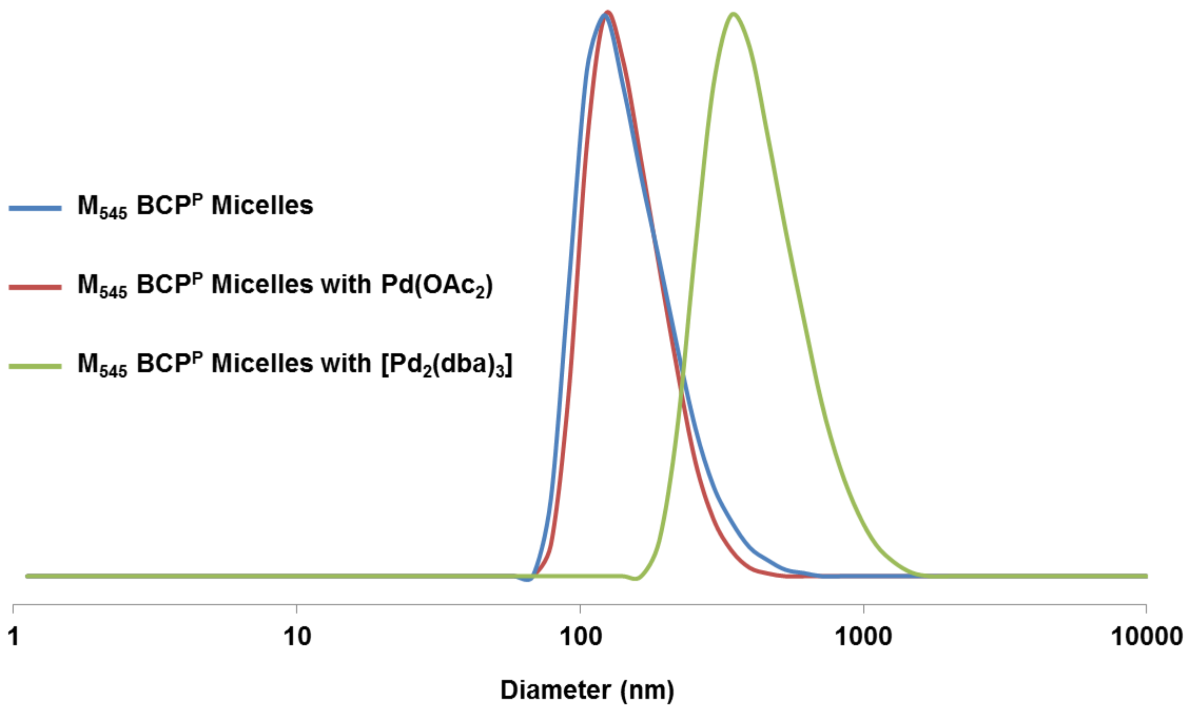

Supplementary Figure 10. Graph showing DLS size distribution by volume of solutions of $\mathbf{M_{545}}$ $\mathbf{BCP^P}$ micelles in EtOAc before, or 1 week after, the addition of 0.25 equiv. of a Pd species.

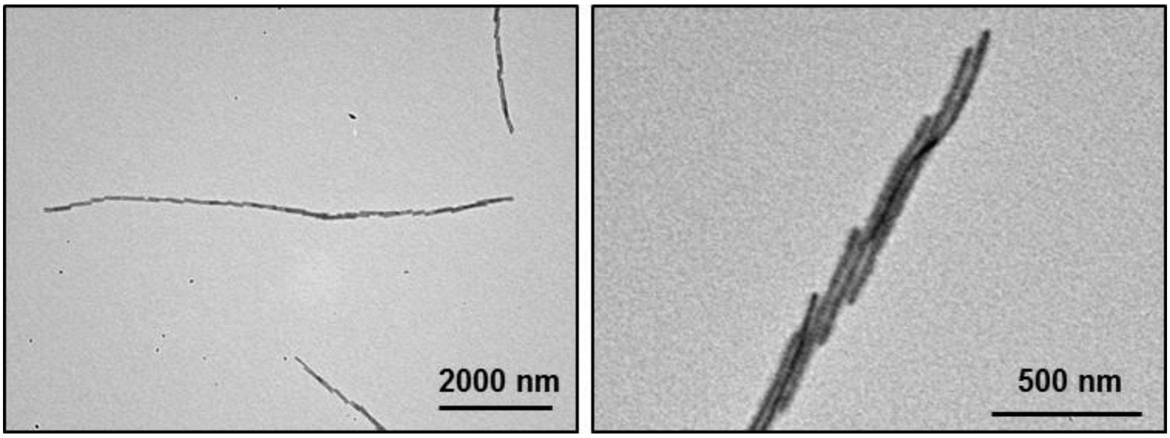

Supplementary Figure 11. TEM images of linear fibres of $\mathbf{M_{545}}$ in EtOAc (1 mg mL^{-1}) after $Pd_2(dba)_3$ addition and further dilution with EtOAc.

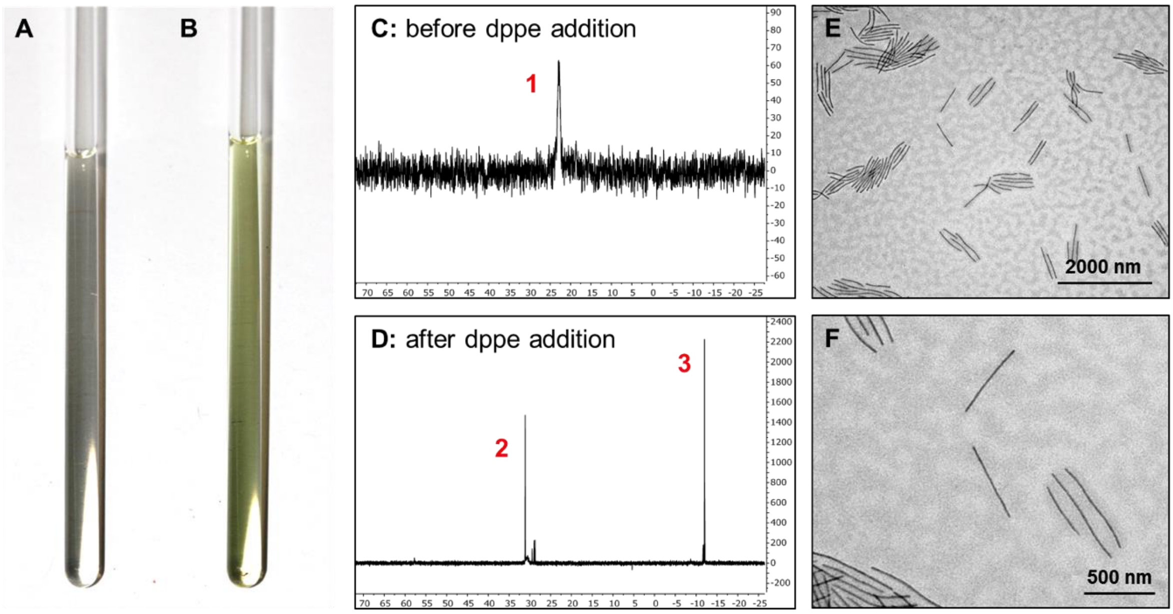

Supplementary Figure 12. Photographic image of 0.5 mg mL^{-1} EtOAc solutions of coordinated $\mathbf{M_{545}}$ micelles before (**A**) and after (**B**) the addition of 1,2-bis-diphenylphosphinoethane (dppe). Corresponding ^{31}P NMR (202 MHz; EtOAc) spectra before (**C**) and after (**D**) the addition of dppe (**BCP^{P}** (**1**), Pd(dppe)$_2$ (**2**), and free dppe (**3**)). **E-F**) TEM images of a drop-cast solution of coordinated $\mathbf{M_{545}}$ micelles in EtOAc after the addition of dppe.

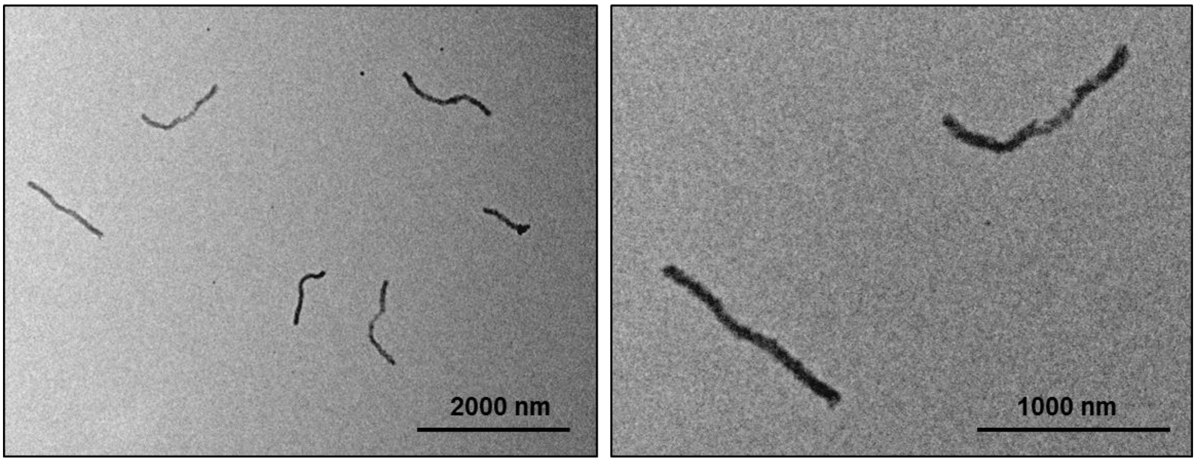

Supplementary Figure 13. TEM images of monodisperse **BCP^{P}** micelles dispersed in THF, a good solvent for both blocks, after intramicelle crosslinking with $Pd_2(dba)_3$ (0.5 equiv. Pd(0)) at a micelle concentration of 0.05 mg mL^{-1}.

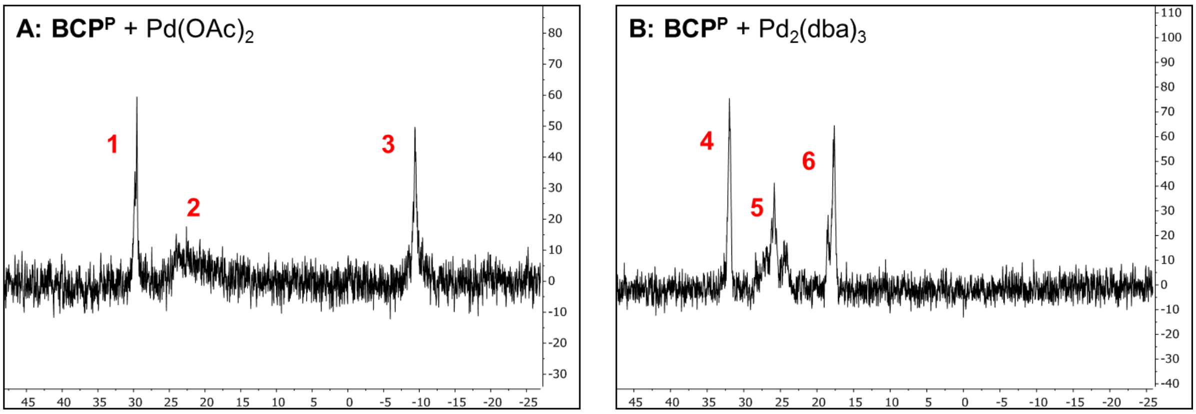

Supplementary Figure 14. ^{31}P NMR (202 MHz; C_6D_6) spectra of **BCP^P** unimer after the addition of 0.5 equiv. Pd as $Pd(OAc)_2$ (**A**) or $Pd_2(dba)_3$ (**B**). Assignments: **1-2** (Pd(**BCP^P**)$_2$(OAc)$_2$ and Pd(L)(**BCP^P**)(OAc)$_2$, where L would most likely correspond to free vinyl groups along the polymer chain or bridging acetate ligands), **3** (free **BCP^P**), **4-6** (Pd(**BCP^P**)$_3$, Pd(dba)(**BCP^P**)$_2$ and Pd(vinyl)(**BCP^P**)$_2$).

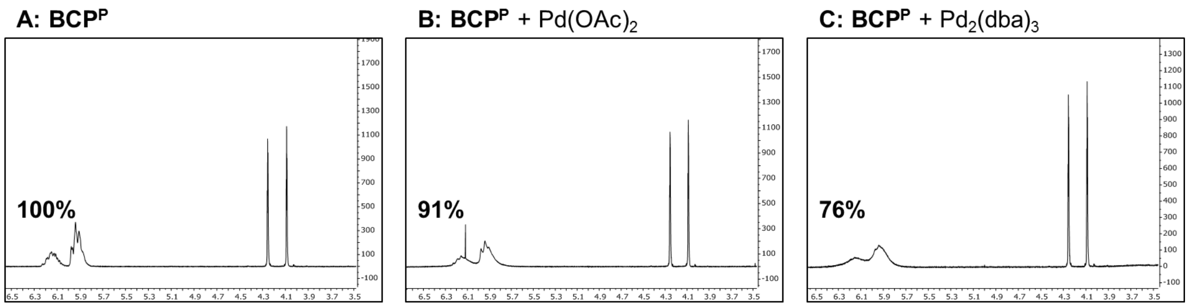

Supplementary Figure 15. ^{1}H NMR (500 MHz; C_6D_6) spectra of the vinyl groups of **BCP$^{\mathbf{P}}$** unimer before (**A**) and after the addition of 0.5 equiv. of $Pd(OAc)_2$ (**B**) or $Pd_2(dba)_3$ (**C**). Signal broadening and reduced integrations of the vinyl groups were observed with increased Pd loadings.

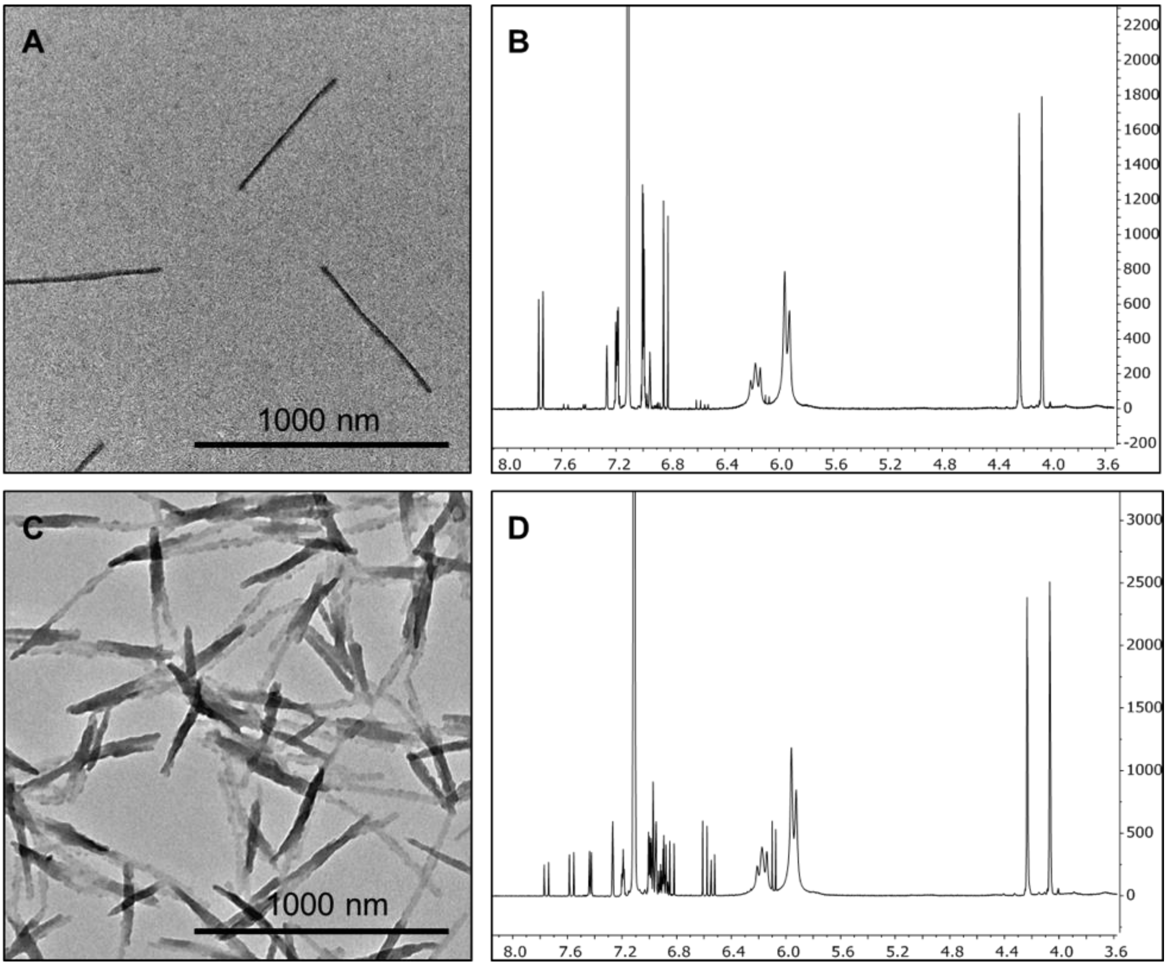

Supplementary Figure 16. TEM images of M(PFS-*b*-PMVS)-*b*-M(PFS-*b*-PDMS)-*b*-M(PFS-*b*-PMVS) block comicelles in EtOAc 1 day (**A**) and 1 week (**C**) after the addition of 0.5 equiv. of $Pd_2(dba)_3$. ^1H NMR (500 MHz; C_6D_6) spectra of the vinyl and aryl groups of PFS-*b*-PMVS unimer 1 day (**B**) and 1 week (**D**) after the addition of $Pd_2(dba)_3$ (0.5 equiv. Pd(0)). No coordination of Pd (increase in micelle electron density) was apparent by TEM after 24 h. However, when samples were left for 1 week, some coordination of Pd was observed, suggestive of a binding mode wherein the vinyl groups of PMVS displace the dba ligands of $Pd_2(dba)_3$ over extended time periods. To obtain comparative data by ^1H NMR, $Pd_2(dba)_3$ was added to a solution of PFS-*b*-PMVS unimers in C_6D_6 (a good solvent for both blocks). After 1 week, ^1H NMR signals corresponding to new environments for dba in solution were observed by ^1H NMR (e.g., δ 6.8-7.6 ppm) when comparing spectra B and D. Analogous TEM and ^1H NMR experiments with $Pd(OAc)_2$ showed no evidence for any reaction.

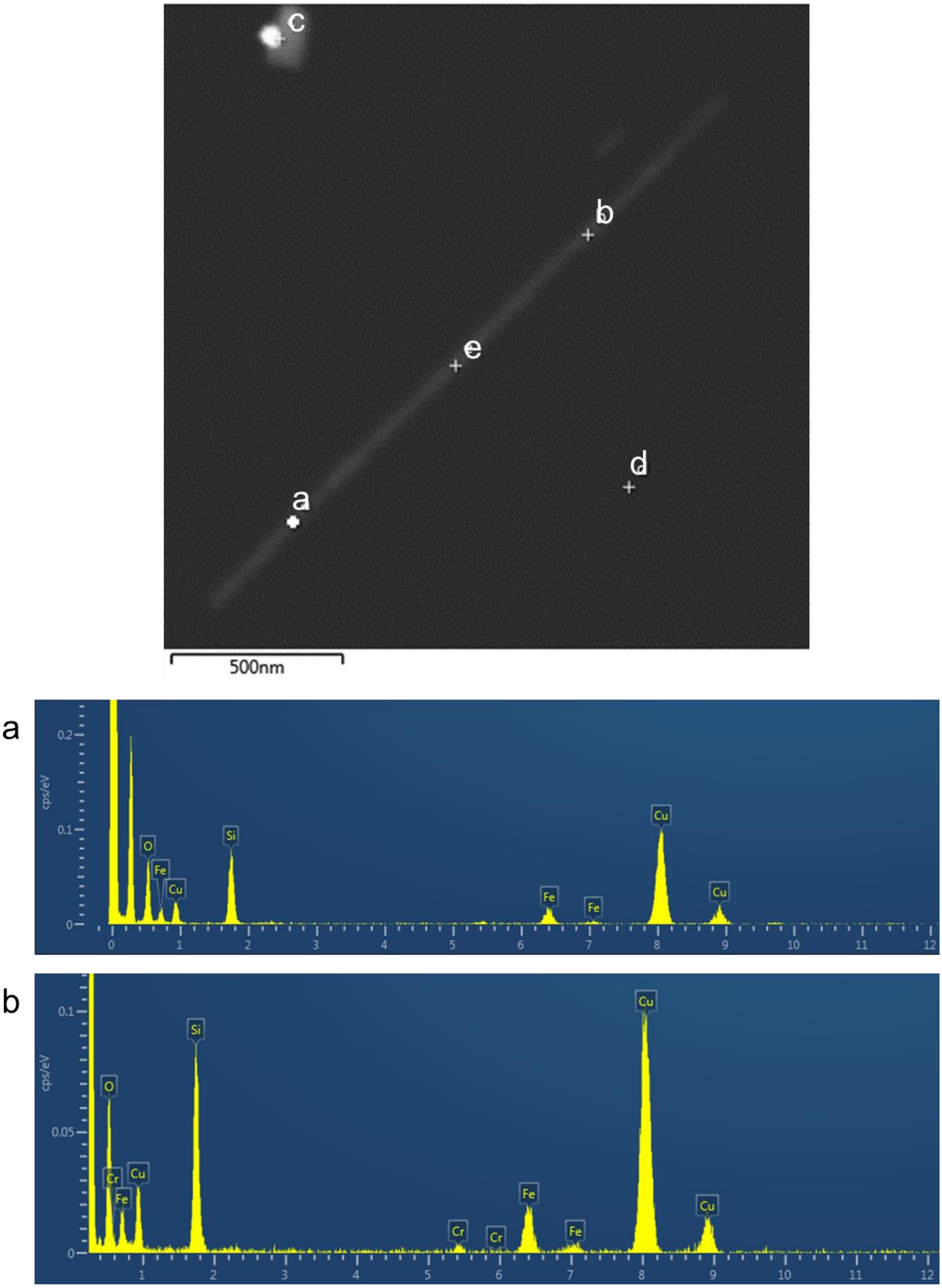
c
b
e
d
a
500nm
a
cps/eV
O
Fe
Cu
Si
Fe
Fe
Cu
Cu
b
cps/eV
O
Cr
Fe
Cu
Si
Cr
Cr
Fe
Fe
Cu
Cu

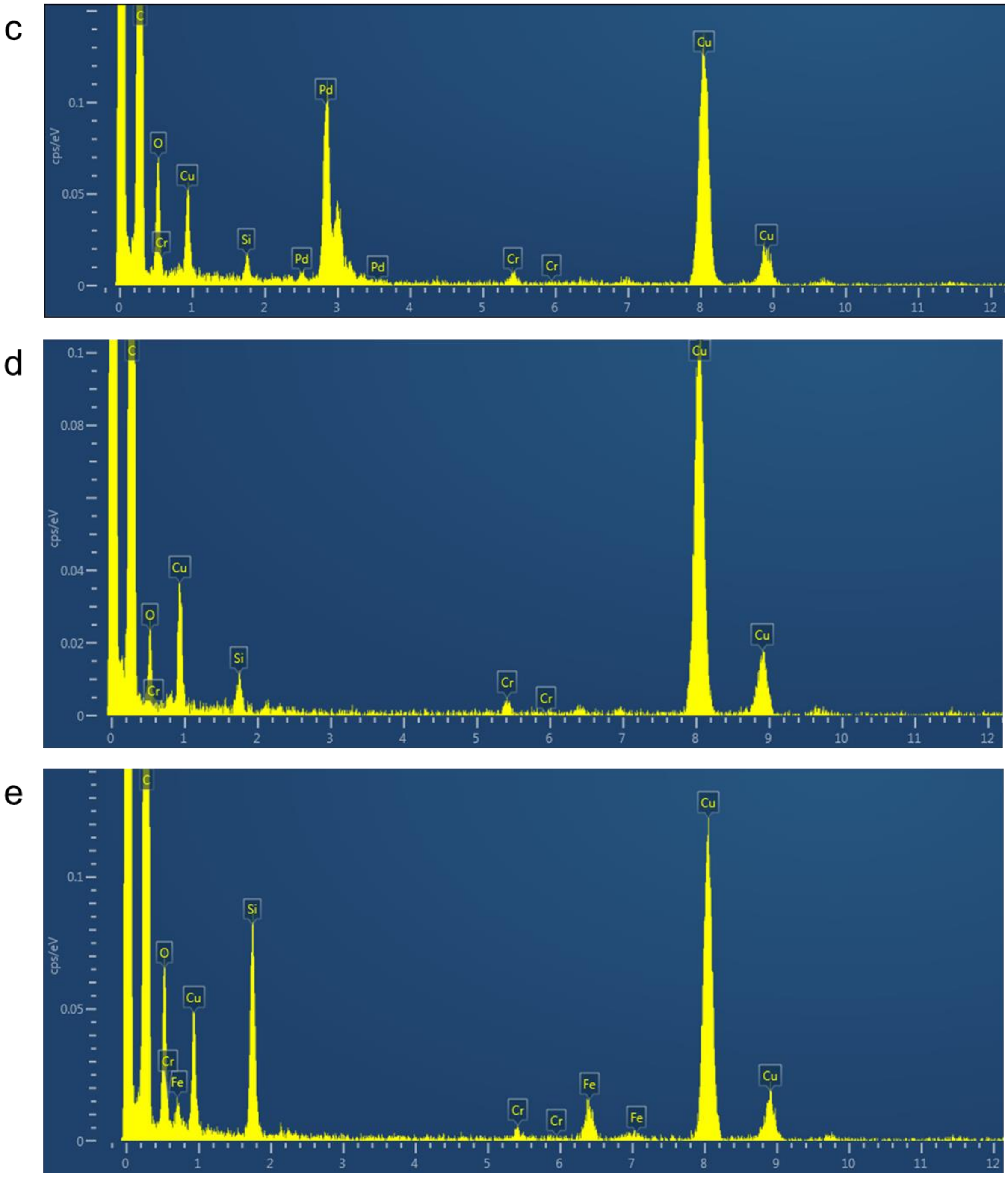

Supplementary Figure 17. Dark-field TEM image and (**a-e**) EDX analysis (at specific locations noted in TEM image) of PFS-*b*-PDMS micelles in EtOAc after addition of $Pd_2(dba)_3$. This control experiment was performed to test whether Pd coordination would be detected to micelles with no phosphine groups. The results should be compared to those for **BCP^{P}** micelles in **Supplementary Fig. 9**. Pd was only detected within aggregates on the substrate in (c). Importantly, no Pd was detected on the micelles in (a), (b) or (e). The target area for EDX analysis (~35 nm diameter).

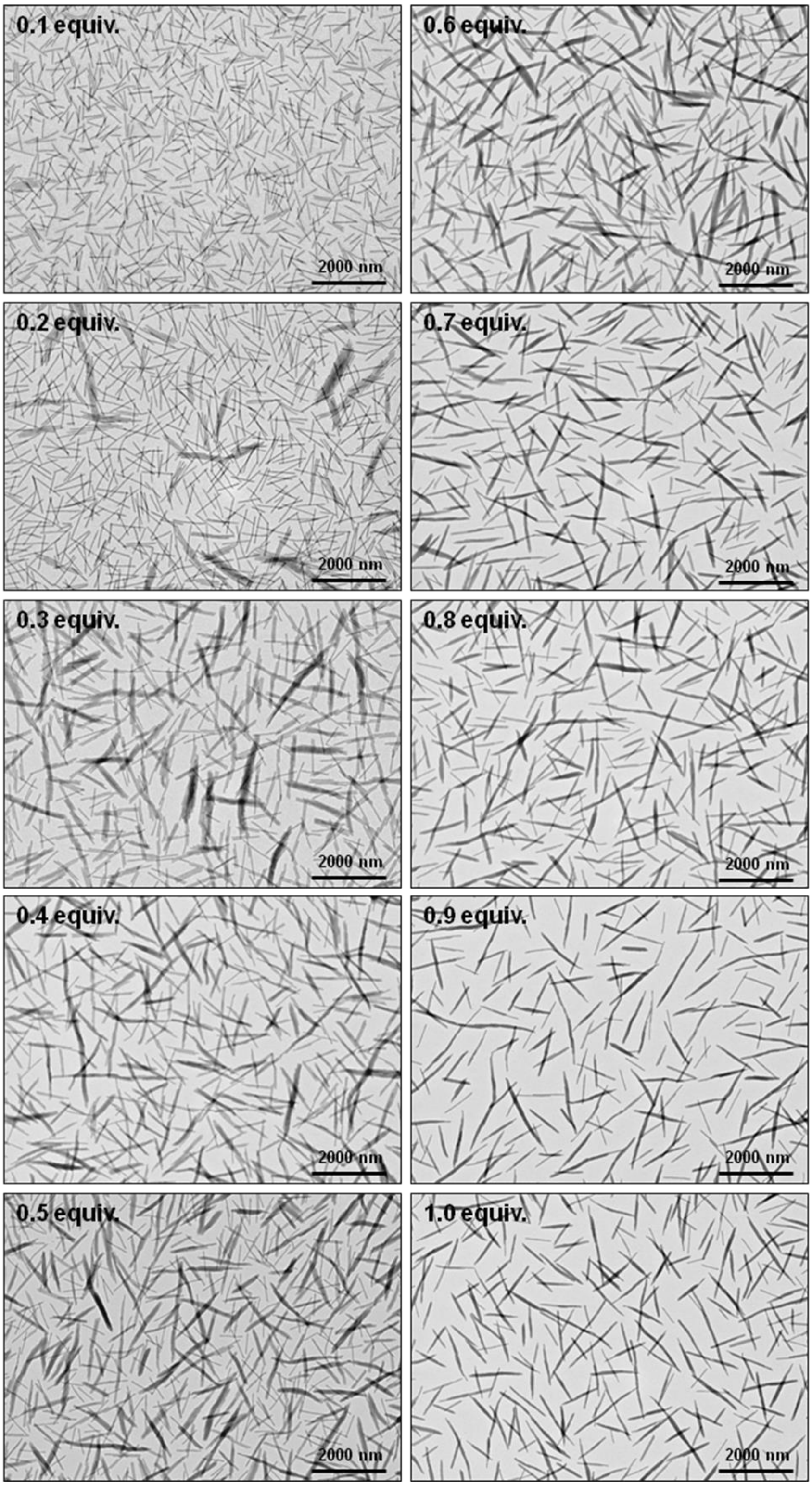

Supplementary Figure 18. TEM images of $\mathbf{M_{545}}$ micelles in EtOAc (0.5 mg mL^{-1}) 24 h after the addition of different equivalents of Pd(0) as $Pd_2(dba)_3$. See **Supplementary Fig. 21** for a graph of Pd(0) equiv. versus mean fibre length (L_n).

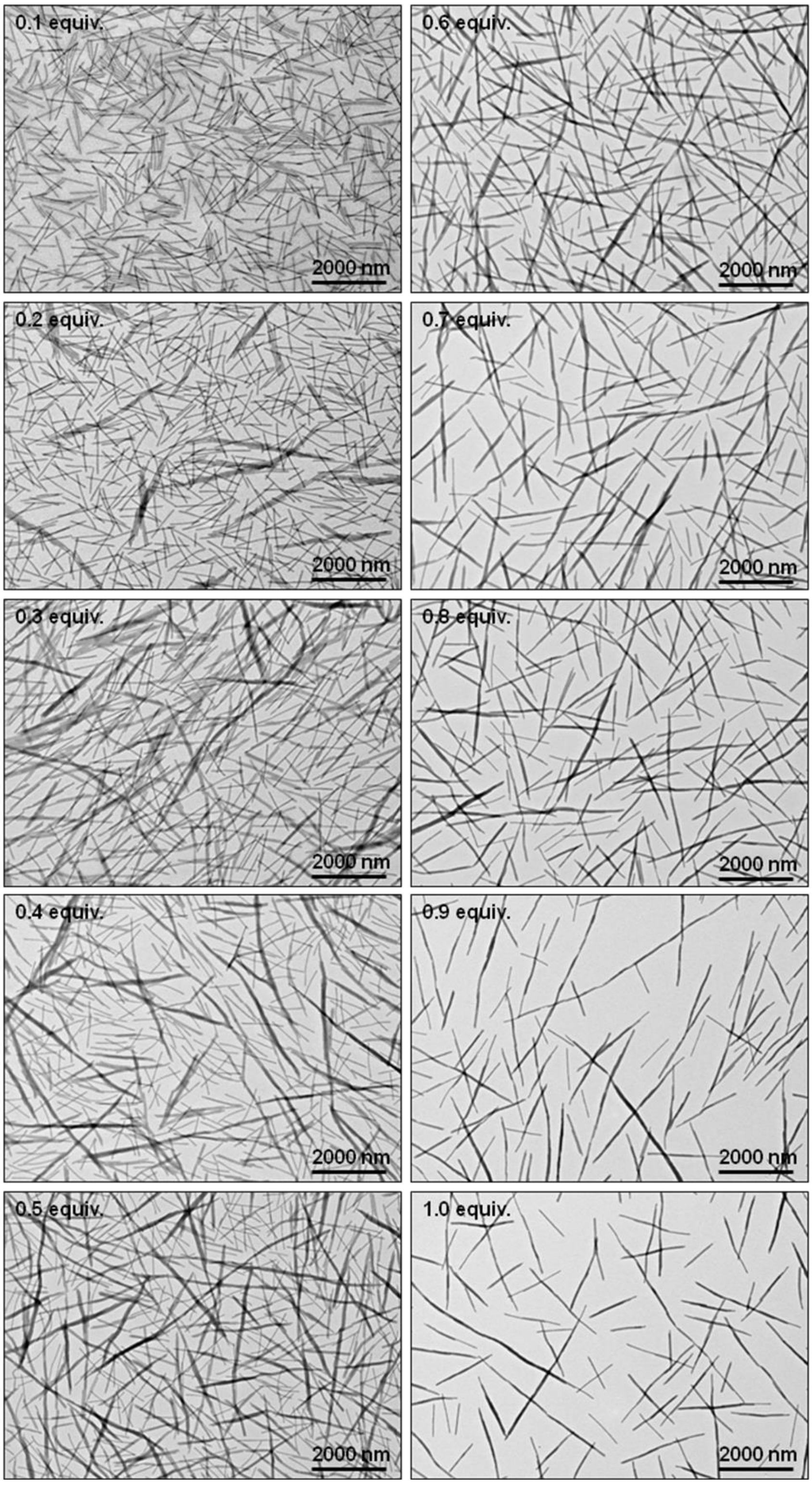

Supplementary Figure 19. TEM images of $\mathbf{M_{970}}$ micelles in EtOAc (0.5 mg mL^{-1}) 24 h after the addition of different equivalents of Pd(0) as $Pd_2(dba)_3$. See **Supplementary Fig. 21** for a graph of Pd(0) equiv. versus mean fibre length (L_n).

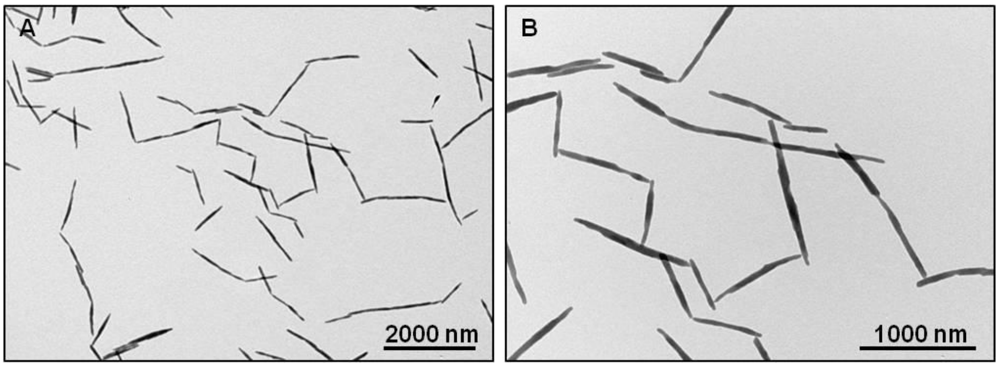

Supplementary Figure 20. TEM images of $\mathbf{M_{545}}$ $\mathbf{BCP^{P}}$ micelles in EtOAc (0.5 mg mL^{-1}) 24 h after addition of $Pd_2(dba)_3$ (1.5 equiv. Pd(0)). As well as linear aggregation, end-to-end aggregation and network formation is also observed which results in the precipitation of larger aggregates. This observation is suggestive of a different mode of intermicelle crosslinking, or a change in the inherent solubility of the micelles.

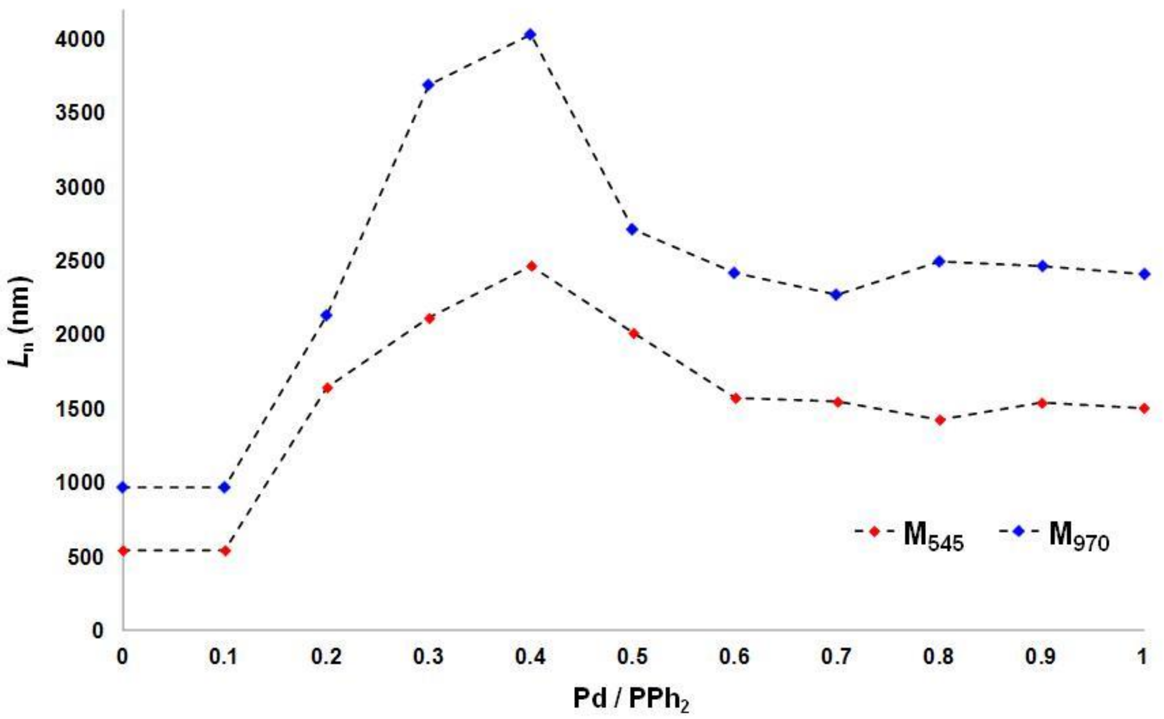

Supplementary Figure 21. Graph of Pd(0) equiv. versus mean fibre length (L_n) for the coordination-driven hierarchical self-assembly of cylindrical **BCP^P** micelle subunits **M_{545}** (L_n = 545 nm, L_w = 570 nm, L_n/L_w = 1.05, σ = 0.03) and **M_{970}** (L_n = 970 nm, L_w = 1005 nm, L_n/L_w = 1.04, σ = 0.02) in EtOAc at a concentration of 0.5 mg mL^{-1}.

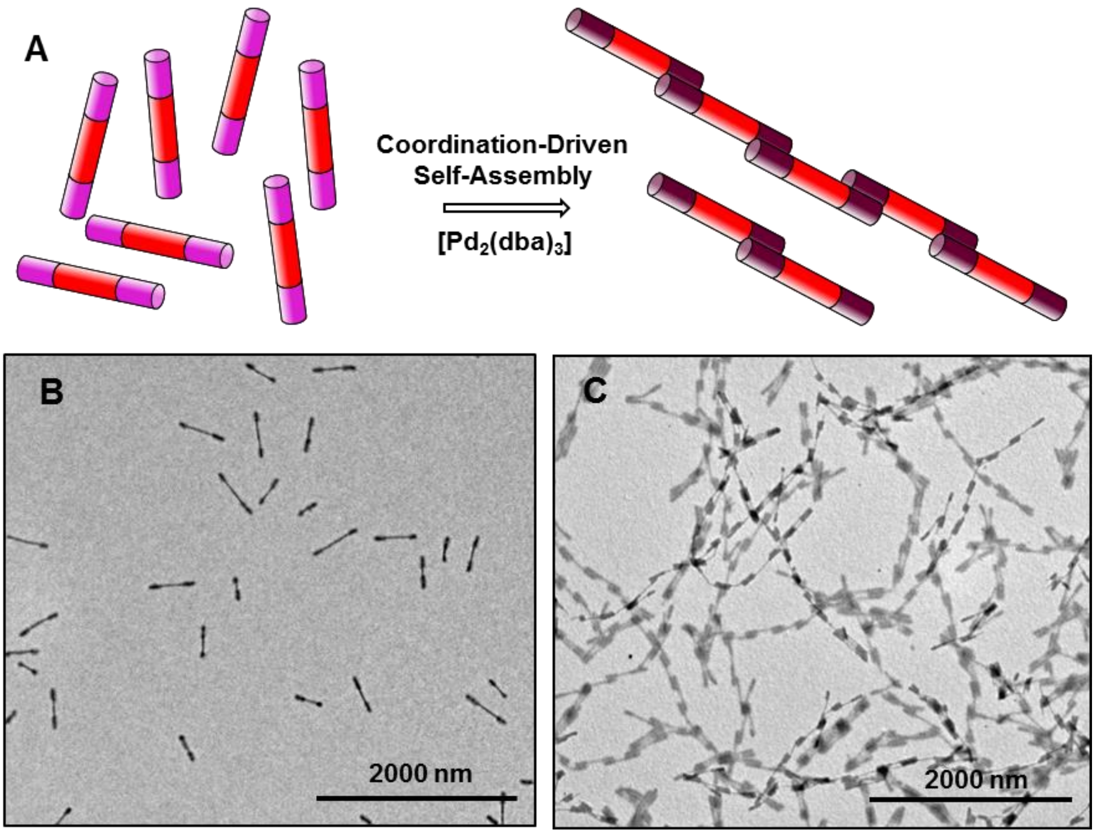

Supplementary Figure 22. Illustrative depiction of the coordination-driven self-assembly of A-B-A triblock comicelles (where A = **BCP^P** and B = PFS_{63}-*b*-$PDMS_{513}$). Red, pink and purple blocks correspond to PFS-*b*-PDMS, **BCP^P** and Pd-coordinated **BCP^P**, respectively. **B**) TEM image of a dilute EtOAc solution (0.01 mg mL^{-1}) of A-B-A triblock comicelles after the addition of Pd_2dba_3 (0.4 equiv. Pd(0)). **C**) TEM image of A-B-A triblock comicelles in EtOAc (0.5 mg mL^{-1}) after the addition of Pd_2dba_3 (0.4 equiv. Pd(0)).

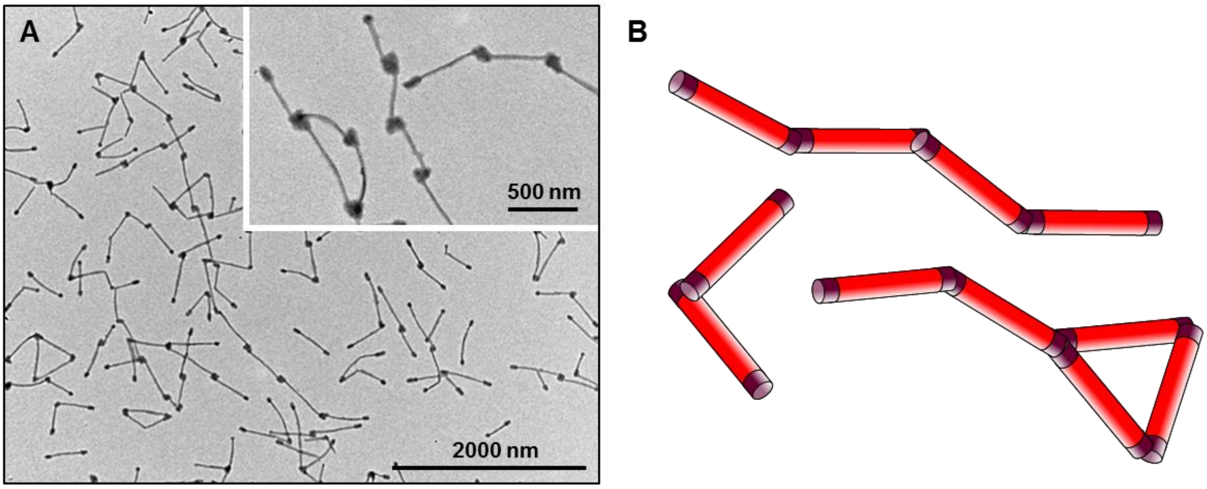

Supplementary Figure 23. A) TEM images of A-B-A triblock comicelles in EtOAc (0.5 mg mL^{-1}) after the addition of Pd_2dba_3 (0.4 equiv. Pd(0)) (where A = $\mathbf{BCP^P}$ and B = PFS_{63}-*b*-$PDMS_{513}$). **B**) Illustrative depiction of coordinated A-B-A triblock comicelles. Red and purple blocks correspond to PFS-*b*-PDMS and Pd-coordinated $\mathbf{BCP^P}$, respectively.

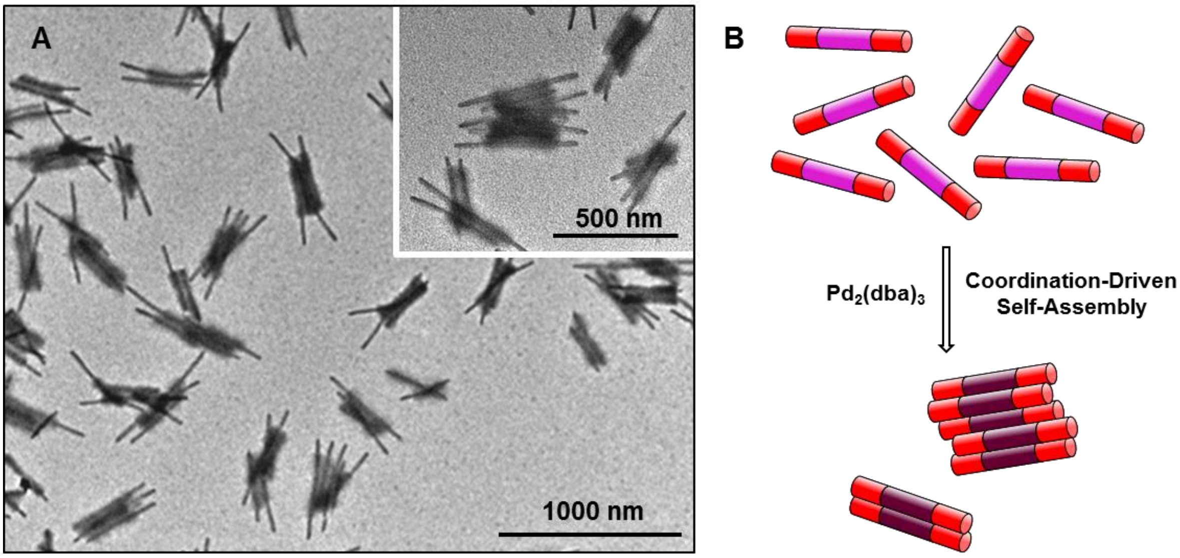

Supplementary Figure 24. A) TEM images of B-A-B triblock comicelles in EtOAc (0.5 mg mL^{-1}) after the addition of Pd_2dba_3 (0.4 equiv. Pd(0)) (where A = $\mathbf{BCP^P}$ and B = PFS_{63}-*b*-$PDMS_{513}$). **B**) Illustrative depiction of the coordination-driven self-assembly of B-A-B triblock comicelles. Red, pink and purple blocks correspond to PFS-*b*-PDMS, $\mathbf{BCP^P}$ and Pd-coordinated $\mathbf{BCP^P}$, respectively.

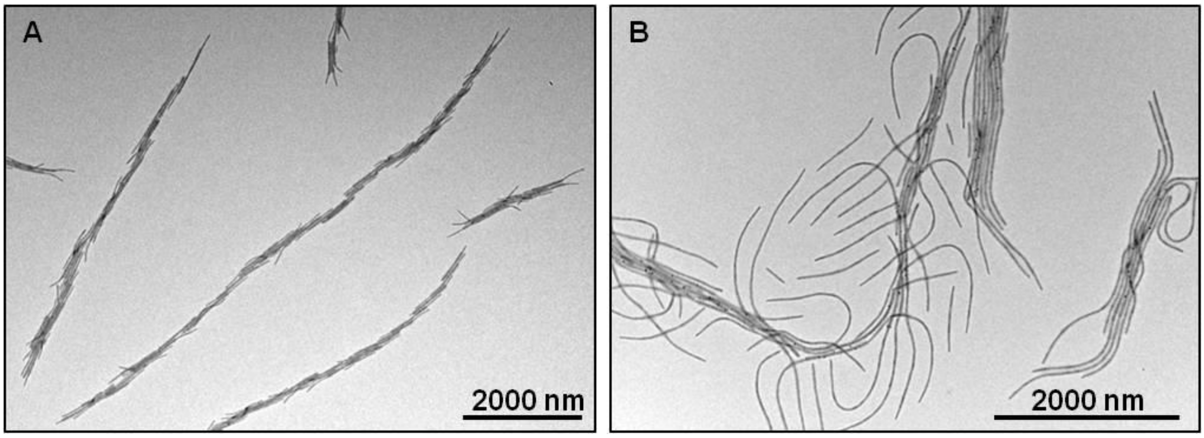

Supplementary Figure 25. TEM images of linear **BCP^{P}** micelle aggregates in EtOAc after addition of **A**) 1 mol equiv. PFDMS$_{63}$-*b*-PDMS$_{513}$ unimer and **B**) 10 mol equiv. PFDMS$_{63}$-*b*-PDMS$_{513}$ unimer (some fragmented micelles can be observed).

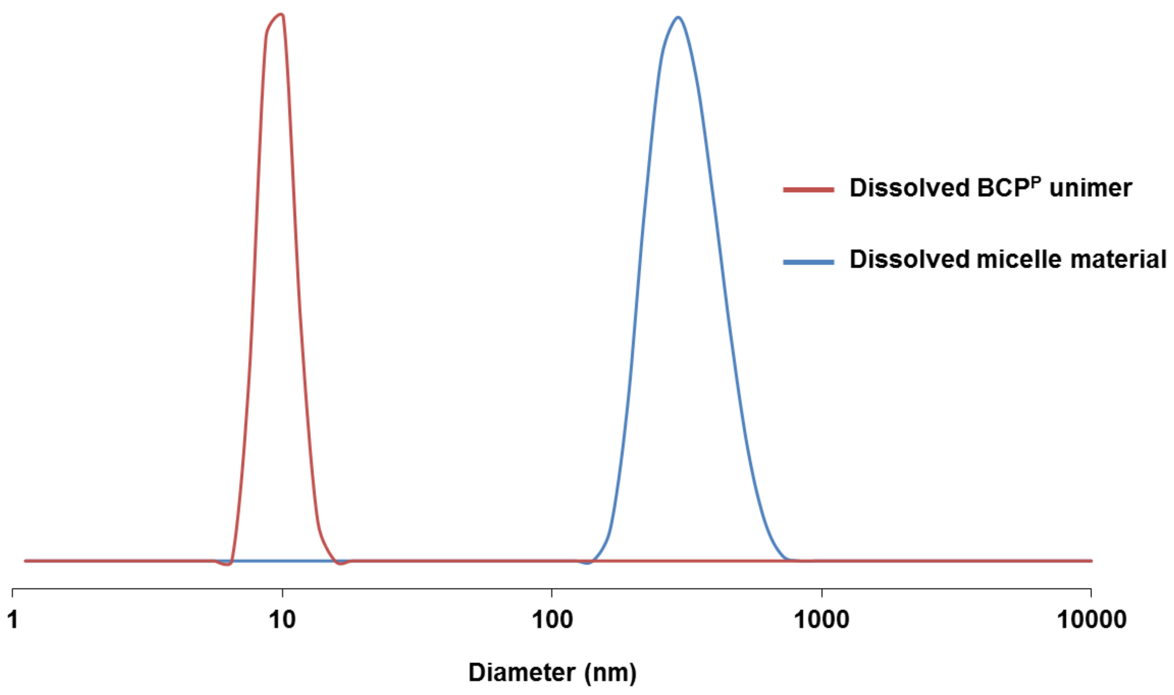

Supplementary Figure 26. Graph showing DLS size distribution by volume of **BCP^{P}** unimer and micelle-based material dissolved in C_6D_6 (1 mg mL^{-1}).

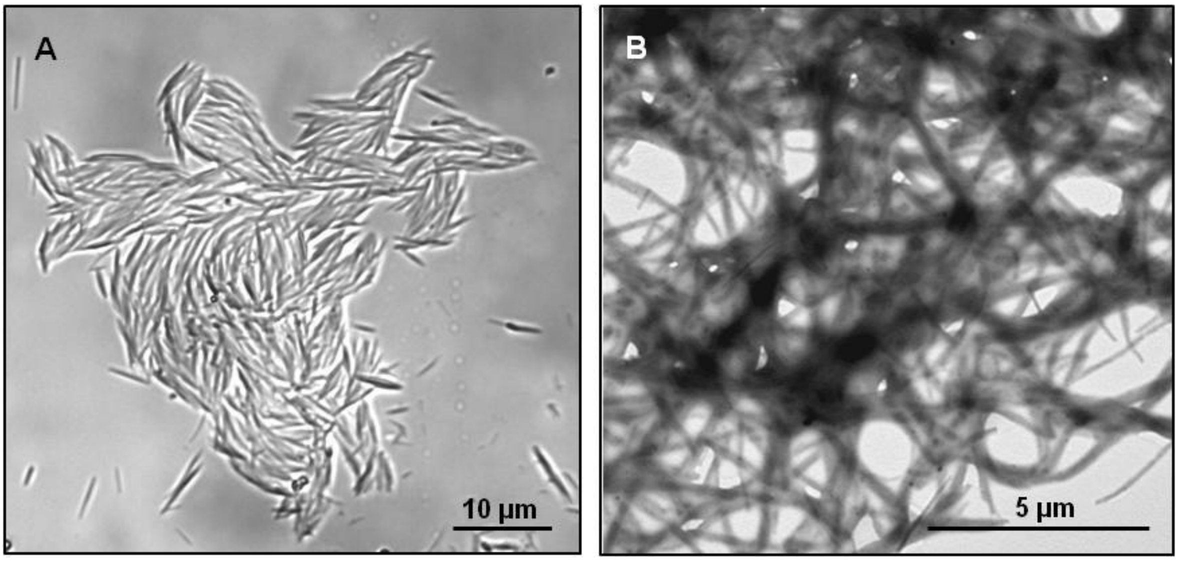

Supplementary Figure 27. Solution optical microscopy image (A) and TEM image (B) of dispersed aggregates of M_{970} (2 mg mL^{-1}) in EtOAc 24 h after the addition of $Pd_2(dba)_3$ (0.4 equiv. Pd(0)).

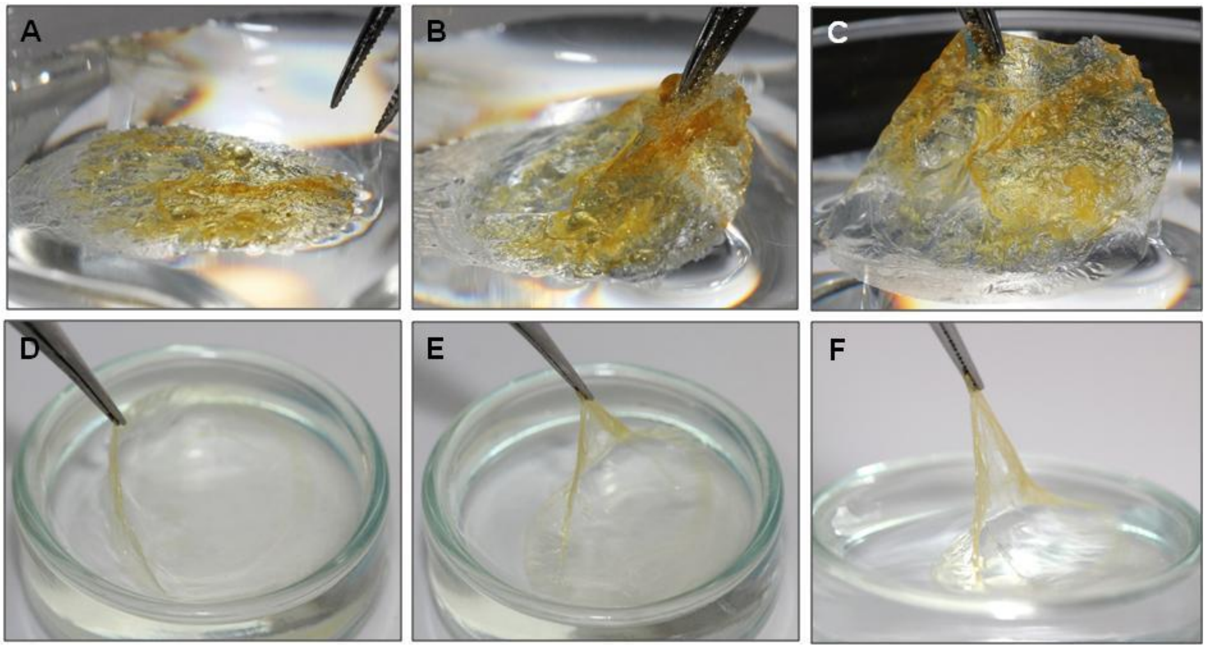

Supplementary Figure 28. Photographic images of micelle films of different thickness on water surfaces. **A-C**) 300 mm diameter film comprised of 3 mg of $\mathbf{BCP^{P}}$ micelles. **D-F**) 300 mm diameter film comprised of 1 mg of $\mathbf{BCP^{P}}$ micelles.

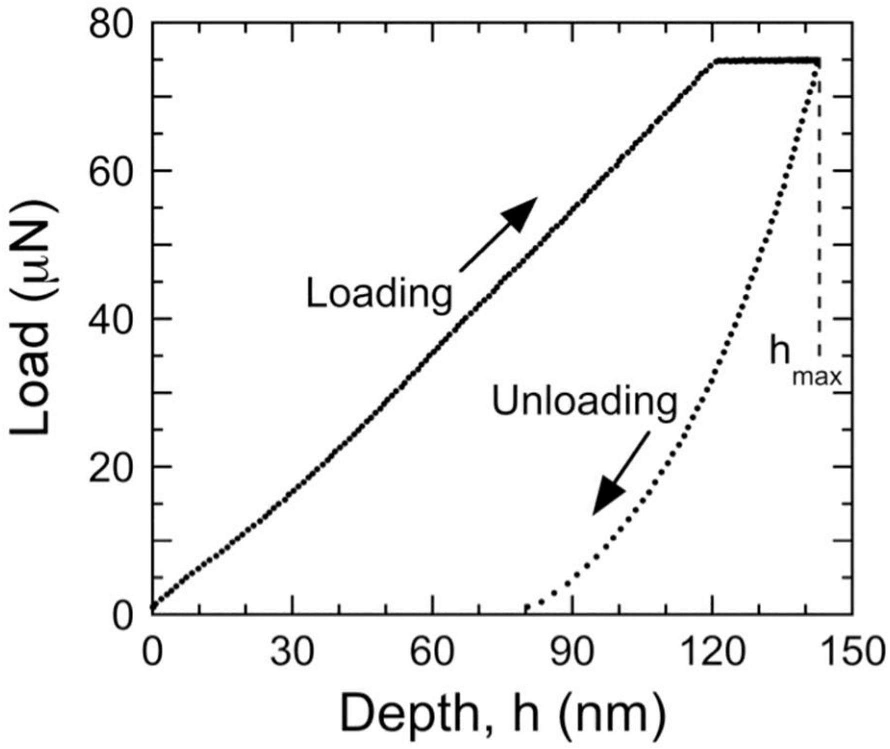

Supplementary Figure 29. Nanoindentation measurements from a loading/unloading cycle on a drop-cast sample of the solution of linear fibres of coordinated $\mathbf{M_{970}}$.

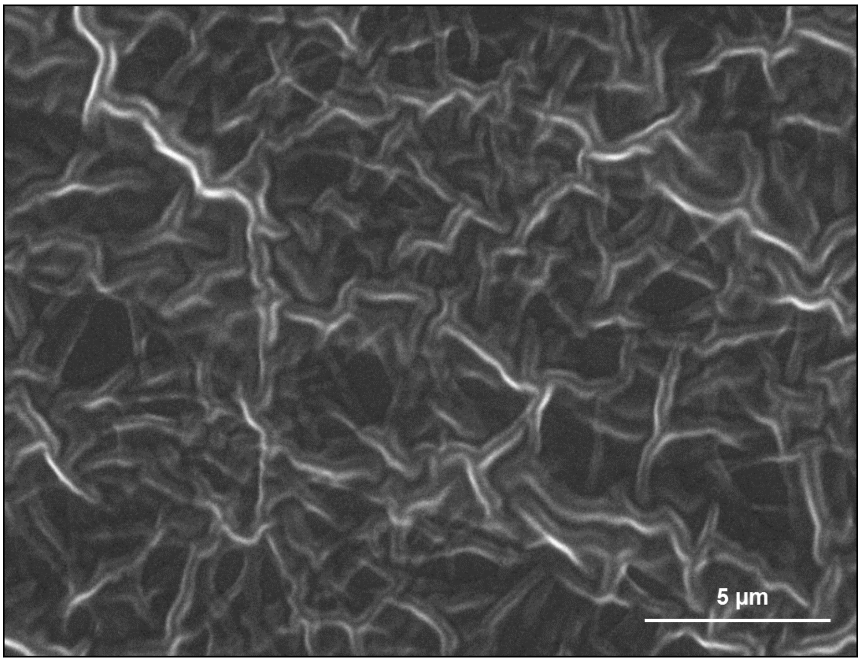

Supplementary Figure 30. SEM image of a dispersed fibrous aggregate of M_{970} micelles in EtOAc (2 mg mL^{-1}), 24 h after the addition of $Pd_2(dba)_3$ (0.4 equiv. Pd(0)).

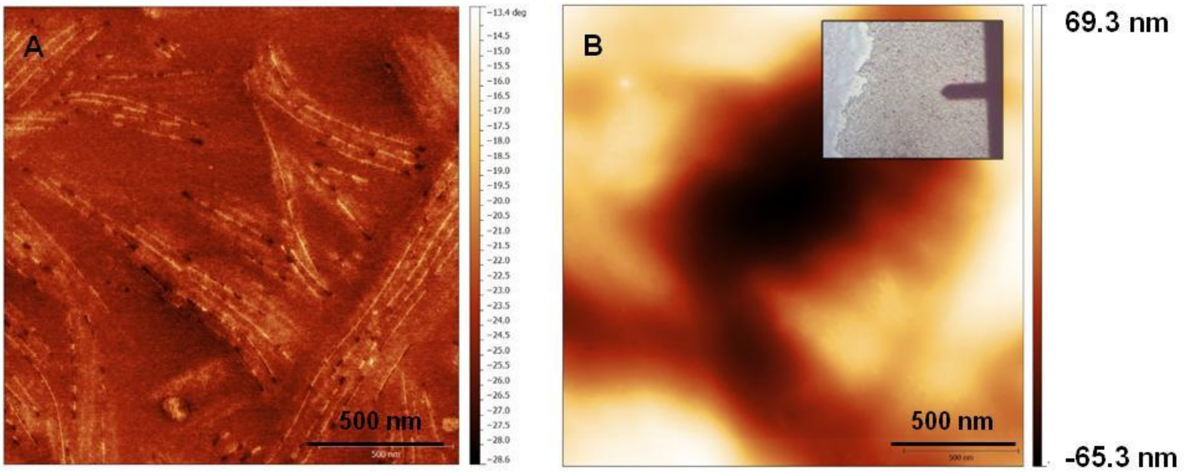

Supplementary Figure 31. AFM phase (**A**) and height (**B**) images of a dispersed fibrous aggregate of M_{970} in EtOAc (2 mg mL^{-1}), 24 h after the addition of $Pd_2(dba)_3$ (0.4 equiv. Pd(0)). Inset shows optical microscope image of sample area analysed by AFM.

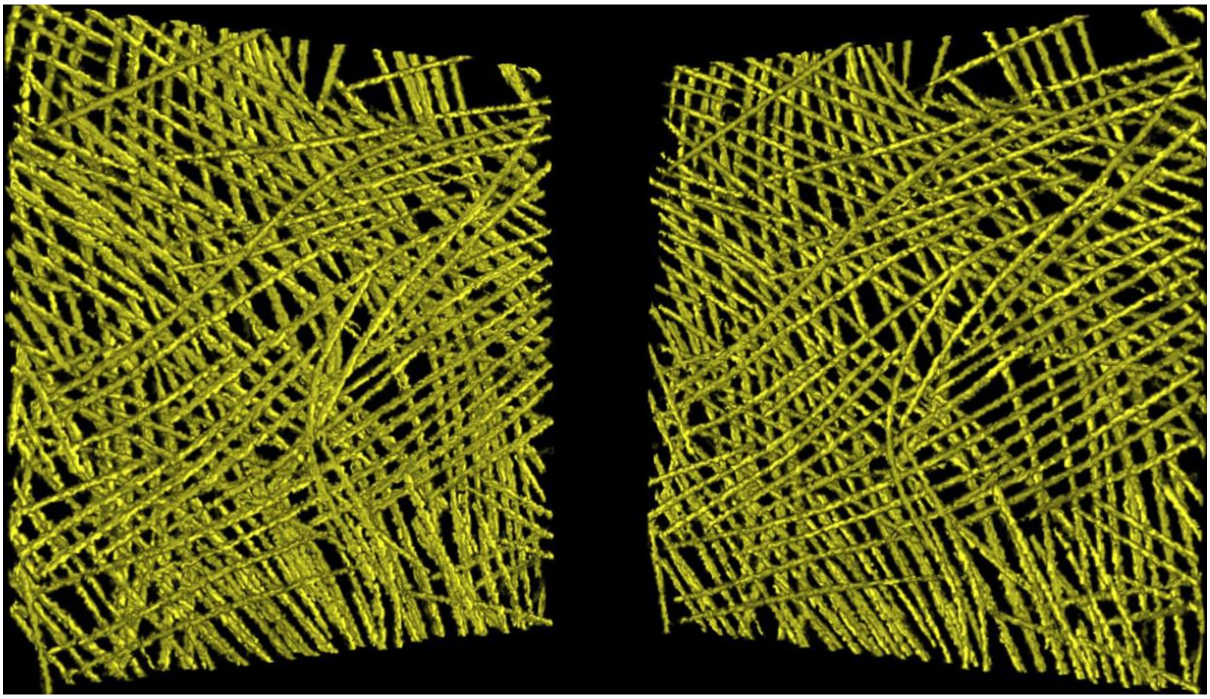

Supplementary Figure 32. Rendered 3D **i**mages (±30° tilts of **Fig. 5f**) from TEMT analysis of a drop-cast sample of linear fibres of coordinated $\mathbf{M_{970}}$ in EtOAc.

Supplementary tables

Supplementary Table 1. Polymer characterisation.

**Polymer**	**DP_n[a)]**	**M_n ($g \cdot mol^{-1}$)**	**PDI[b)]**
$PFDMS_{63}$-*b*-$PDMS_{513}$	-	53 400[c)]	1.14
$PFDMS_{68}$-*b*-$PMVS_{670}$	-	74 300[c)]	1.18
$PFDMS_{61}$ Aliquot	61	14 769[a)]	1.04
$PFDMS_{61}$-*b*-$PMVS_{574}$	-	64 300[c)]	1.17
Hydrophosphinated $PFDMS_{61}$-*b*-$PMVS_{574}$	-	101 800[c)]	1.36[d)]

[a)]Determined by MALDI-TOF. [b)]Determined by GPC with multi-detector or conventional calibration using polystyrene standards (**Supplementary Fig. 1**). [c)]Determined by ^{1}H NMR integration of the vinyl protons (3H) of PMVS and the methyl protons (6H) of PFDMS. [d)]After sulfurisation of pendant phosphines.

Supplementary Table 2. Statistical length analysis for the growth of $\mathbf{BCP^P}$ micelles.

m_{unimer} / m_{seed}	L_n (nm)	L_w (nm)	L_w/L_n
0[a)]	37	43	1.16
5	210	218	1.04
10	351	362	1.03
20	625	643	1.03
40	1229	1249	1.02

[a)]For seed micelles prepared by sonication (**see Supplementary Fig. 3**).

Supplementary Table 3. Statistical length analysis for linear fibres of **BCP**[P] micelles.

Starting BCP^{P} micelles	Pd(0) equiv.	L_n (nm)	L_w (nm)	L_w/L_n
$\mathbf{M_{545}}$	0.1 [a)]	n/a	n/a	n/a
$\mathbf{M_{545}}$	0.2	1645	2000	1.21
$\mathbf{M_{545}}$	0.3	2115	2575	1.22
$\mathbf{M_{545}}$	0.4	2470	2910	1.18
$\mathbf{M_{545}}$	0.5	2010	2325	1.15
$\mathbf{M_{545}}$	0.6	1575	1825	1.16
$\mathbf{M_{545}}$	0.7	1550	1820	1.18
$\mathbf{M_{545}}$	0.8	1430	1680	1.18
$\mathbf{M_{545}}$	0.9	1540	1720	1.11
$\mathbf{M_{545}}$	1.0	1510	1750	1.16
$\mathbf{M_{970}}$	0.1[a)]	n/a	n/a	n/a
$\mathbf{M_{970}}$	0.2	2130	2380	1.12
$\mathbf{M_{970}}$	0.3	3690	5005	1.36
$\mathbf{M_{970}}$	0.4	4030	4980	1.24
$\mathbf{M_{970}}$	0.5	2420	3330	1.38
$\mathbf{M_{970}}$	0.6	2710	3490	1.29
$\mathbf{M_{970}}$	0.7	2270	2980	1.31
$\mathbf{M_{970}}$	0.8	2495	3245	1.30
$\mathbf{M_{970}}$	0.9	2465	3105	1.26
$\mathbf{M_{970}}$	1.0	2415	2995	1.24

[a)]No coordination-driven self-assembly was observed.

For representative TEM images see **Supplementary Fig. 18 and 19**.

Supplementary note 1

As the micelles in this study are kinetically trapped due to crystallization of the core, there is no unimer-micelle equilibrium. In the ^{31}P NMR experiments, we are therefore measuring the palladium coordinated to the phosphines within the micelles (**Supplementary Fig. 5**). For comparative ^{31}P NMR experiments on the molecularly dissolved **BCP^{P}** (in a good solvent for both blocks) see **Supplementary Fig. 14**.

EDX analysis of **BCP^{P}** micelles after the addition of $Pd_2(dba)_3$ only detected Pd on the micelles and not on the background substrate (**Supplementary Fig. 9**). No coordination of $Pd_2(dba)_3$ to the micelles was detected when only PFS-*b*-PDMS micelles were used (**Supplementary Fig. 17**).

In terms of the actual micelle size, a contraction is expected due to intramicelle crosslinking in the micelle coronas. However, by TEM, a slight increase in size is observed due to the increased electron density of Pd which allows visualisation of the previously unobservable polysiloxane corona.

For the coordination-driven self-assembly of A-B-A micelles (where A = **BCP^{P}** and B = PFS-*b*-PDMS) see **Supplementary Fig. 22 and Fig. 23**. For the coordination-driven self-assembly of B-A-B micelles see **Supplementary Fig. 24**. The stoichiometry of Pd(0) (0.4 equiv.) used for these experiments was chosen to maximise the length of the resulting fibers and was based on the results from the experiments using pure **BCP^{P}** micelles (see **Fig. 3d**).

When $Pd_2(dba)_3.CHCl_3$ was added in the presence of air, or when using non-degassed solvents, no coordination-driven self-assembly was observed by TEM. Furthermore, when an excess of dba was added into the micelle solution before $Pd_2(dba)_3$ addition, no significant change in the coordination-driven self-assembly was observed by TEM. The same coordination-driven self-assembly was observed when $Pd(dba)_2$ was used instead of $Pd_2(dba)_3 \cdot CHCl_3$.
